# Overlap of nonbreeding wandering albatrosses with fisheries and implications for colony‐specific population trajectories at South Georgia

**DOI:** 10.1111/cobi.70260

**Published:** 2026-03-23

**Authors:** V. Warwick‐Evans, E. J. Pearmain, R. A. Phillips

**Affiliations:** ^1^ British Antarctic Survey Natural Environment Research Council Cambridge UK; ^2^ Department of Zoology University of Cambridge Cambridge UK

**Keywords:** fisheries bycatch, fisheries overlap, foraging ecology, pelagic seabirds, population decline, population overlap, Aves marinas pelágicas, captura incidental en la pesca, ecología alimentaria, disminución de la población, superposición de pesquerías, superposición de poblaciones, **∖**渔业兼捕, 渔业重叠, 觅食生态学, 种群数量下降, 种群重叠, 远洋海鸟

## Abstract

Bycatch in fisheries is one of the most serious threats to pelagic seabirds, causing major population declines. Mitigation measures can reduce bycatch substantially, but many fisheries fail to apply best practices, and seabird mortality remains high. Seabirds often segregate at sea according to sex and life‐history stage, and bycatch risk can vary accordingly. Few studies have tested whether spatial segregation among colonies in foraging areas affects bycatch risk. We tracked nonbreeding wandering albatrosses (*Diomedea exulans*) from Bird Island and neighboring Prion Island, South Georgia, to investigate whether differences in at‐sea distributions and overlap with fisheries explain the contrasting population trends. Tracked individuals at Bird Island were of known status (immature or nonbreeding adults), and at Prion Island, they were most likely older immatures and potentially a few nonbreeding adults. There was marked spatial segregation between age classes at Bird Island, but the pattern between breeding sites was more complex. The overlap with fisheries was highest in nonbreeding adults from Bird Island, which experienced a faster rate of population decline than at Prion Island, where overlap with fisheries was lower. Overlap was highest with Chinese, South Korean, and Taiwanese squid jiggers, Taiwanese pelagic longliners, and Argentinian and Spanish trawlers. By improving our knowledge of the spatiotemporal overlap of seabirds with fisheries, management initiatives can be directed at the fleets that represent the greatest threats.

## Abstract (es)

Superposición de albatros errantes no reproductores con las pesquerías y sus implicaciones para las trayectorias poblacionales específicas de las colonias en Georgia del Sur

**Resumen:**

Las capturas incidentales en la pesca son una de las amenazas más graves para las aves marinas pelágicas, ya que provocan importantes descensos en sus poblaciones. Las medidas de mitigación pueden reducir considerablemente las capturas incidentales, pero muchas pesquerías no aplican las mejores prácticas y la mortalidad de las aves marinas sigue siendo elevada. Las aves marinas suelen segregarse en el mar según su sexo y etapa del ciclo vital, y el riesgo de captura accidental puede variar en consecuencia. Pocos estudios han analizado si la segregación espacial entre colonias en las zonas de alimentación afecta al riesgo de captura accidental. Hemos realizado un seguimiento de los albatros errantes (*Diomedea exulans*) no reproductores de la isla Bird y la vecina isla Prion, en Georgia del Sur, para investigar si las diferencias en la distribución en el mar y la superposición con las pesquerías explican las tendencias poblacionales contrastadas. Los individuos rastreados en Bird Island tenían un estatus conocido (adultos inmaduros o no reproductores), y en Prion Island probablemente eran inmaduros de mayor edad y posiblemente algunos adultos no reproductores. En Bird Island se observó una marcada segregación espacial entre las clases de edad, pero el patrón entre los lugares de reproducción era más complejo. La superposición con las pesquerías fue mayor en los adultos no reproductores de Bird Island, que experimentaron una tasa de disminución de la población más rápida que en Prion Island, donde la superposición con las pesquerías fue menor. La superposición fue mayor con los pescadores de calamar chinos, surcoreanos y taiwaneses, los palangreros pelágicos taiwaneses y los arrastreros argentinos y españoles. Al mejorar nuestro conocimiento de la superposición espacio‐temporal de las aves marinas con las pesquerías, las iniciativas de gestión pueden dirigirse a las flotas que representan las mayores amenazas.

## 非繁殖期漂泊信天翁与渔业活动的重叠及其对南乔治亚岛特定鸟群种群轨迹的影响

**【摘要】** 渔业兼捕是远洋海鸟面临的最严重威胁之一, 会导致严重的种群数量下降。实施减缓措施可大幅减少兼捕, 但许多渔船未能采用最佳实践, 导致海鸟死亡率居高不下。海鸟在海洋中通常根据性别和生活史阶段而分群活动, 因此兼捕风险也随之变化。目前, 很少有研究检验觅食区内不同鸟群的空间分隔是否影响兼捕风险。本研究追踪了南乔治亚岛鸟岛及邻近普里昂岛非繁殖期的漂泊信天翁(*Diomedea exulans*), 以探究其海上分布差异及与渔业活动的重叠程度是否解释了其种群趋势的差异。我们在鸟岛追踪的个体具有明确状态(亚成体或非繁殖成体), 而普里昂岛的个体则多为年龄较大的亚成体, 可能包含少量非繁殖成体。鸟岛各年龄组间存在显著空间分隔, 但繁殖地间的分布格局更为复杂。鸟岛非繁殖成体与渔业活动重叠度最高, 其种群数量下降的速度快于与渔业重叠度较低的普里昂岛个体。重叠度最高的渔业类型包括:中国、韩国、中国台湾的鱿鱼钓船、中国台湾的远洋延绳钓船、阿根廷及西班牙拖网渔船。通过增进对海鸟与渔业活动时空重叠的认知, 管理措施可精准指向最具威胁性的船队。**【翻译:胡怡思;审校:聂永刚】**

## INTRODUCTION

Seabirds are one of the most threatened groups of birds, with 57% of species declining and 43% listed as globally threatened (Phillips et al., 2023). Their main threats are invasive species at breeding sites, bycatch during fishing operations, and overfishing (Dias et al., 2019; Phillips et al., 2023). Seabirds spend a large proportion of their lifetime at sea, frequently travelling vast distances and consequently encountering numerous fishing fleets (Beal, Dias, et al., 2021; Clay et al., 2019). Seabirds and fisheries exploit similar regions, where topographic and oceanographic features lead to abundant, predictable, and accessible prey (stocks). Fine‐scale interactions occur when seabirds are attracted to fishing vessels in pursuit of offal, discards, and bait (Carneiro, Clark, et al., 2022; Tasker et al., 2000). However, seabirds may become entangled or hooked on longlines, strike trawl cables and monitoring wires, or be caught in gillnets (Louzao et al., 2011; Phillips et al., 2024). If used in appropriate combinations, mitigation measures including night setting, heavier line weighting, bird‐scaring (streamer) lines, and offal management are effective in reducing bycatch to negligible levels (Collins et al., 2021; Phillips et al., 2016). However, many fisheries fail to operate best practices, and bycatch remains at unsustainably high levels for many seabirds, leading to substantial population declines.

Albatrosses and large petrels are at particular risk from bycatch because they are long‐lived, with delayed maturity and slow reproductive rates, and therefore reduced adult and juvenile survival has major impacts on population trends (Pardo et al., 2017; Phillips et al., 2016). Among the species that are worst affected is the wandering albatross (*Diomedea exulans*), which is listed as globally vulnerable on the International Union for Conservation of Nature (IUCN) Red List. The population in South Georgia decreased by ∼2% a year between 1983/84 and 2014/15, and by 0.1% a year from then until 2023/24; the main or only driver appears to be fisheries bycatch, particularly in the Atlantic Ocean (Mackley et al., 2025). It is listed as a global high‐priority population for conservation by the Multilateral Agreement on the Conservation of Albatrosses and Petrels (ACAP Advisory Committee, 2011; Poncet et al., 2017). Previous studies have shown that spatial segregation of wandering albatrosses by age and sex affects fisheries overlap and bycatch risk, particularly that females and immatures tend to overlap more with pelagic longline fisheries (Jiménez et al., 2016; Orgeret et al., 2021; Weimerskirch et al., 2014). Lower wing loading enables females to exploit lighter winds, and so they spend more time than males in tropical waters (Shaffer et al., 2001). Juvenile and immature seabirds tend to disperse more widely than adults, as they explore new environments and improve foraging efficiency. Habitat specialization, differential responses to environmental cues, or avoidance of competition may therefore lead them to use suboptimal or more northerly habitats (Clay et al., 2016; Frankish et al., 2020; Phillips et al., 2017; Weimerskirch et al., 2014). This may lead to age biases in bycatch rates (Gianuca et al., 2019) or an interaction between age and sex, for example, in the Indian Ocean, where older male wandering albatrosses are more likely to travel south than younger males (Lecomte et al., 2010; Weimerskirch et al., 2014).

The rate of population decline of wandering albatrosses varies considerably across South Georgia (Mackley et al., 2025; Poncet et al., 2017; Rackete et al., 2021). This appears to be related at least in part to spatial segregation between colonies during the breeding season, which affects overlap with fisheries and hence bycatch risk (Warwick‐Evans et al., 2026). Whether this also applies to nonbreeding birds and contributes to the contrasting population trends is unknown. After fledging, juvenile wandering albatrosses travel north to warmer waters and do not return to the colony for 3–7 years (Croxall et al., 1990; Weimerskirch et al., 2014). When they do, the immatures behave for a period of several weeks as central‐place foragers, then disperse for the rest of the year. After recruitment, wandering albatrosses—if successful—usually alternate a breeding year during which they are central‐place foragers, with a sabbatical year at sea (Tickell, 1968). Immatures and nonbreeding adults from Bird Island may remain resident in the south Atlantic Ocean and Patagonian Shelf, also use the Humbolt Current off the coast of Chile, or migrate long distances to distant foraging grounds in the east Atlantic, Indian, or Pacific oceans, in some cases circumnavigating Antarctica multiple times (Clay et al., 2018; Mackley et al., 2010). Nonbreeding birds therefore overlap with several seabird bycatch hotspots (Clay et al., 2019). To date, all tracking of nonbreeding wandering albatrosses has been at Bird Island, and nothing was known about the distribution and fisheries overlap of nonbreeders from other colonies at South Georgia. The aim of our study was therefore to establish whether spatial segregation and relative overlap with fisheries may contribute to the contrasting trends of wandering albatross populations from Bird Island and Prion Island (∼50 km apart), South Georgia. Birds from Prion Island were unringed and, based on plumage, timing of fieldwork, and subsequent observations, were considered to be mostly older immatures and possibly some nonbreeding adults (i.e., deferring breeders). In order to disentangle the effects of status and breeding site, we quantified fisheries overlap for these birds and for birds of known status (immatures and nonbreeding adults) from Bird Island. We investigated spatial segregation and dispersal of each group in addition to quantifying the spatiotemporal overlap with fishing activity.

## METHODS

All fieldwork was approved by the British Antarctic Survey Animal Welfare and Ethical Review Body and carried out under permit from the Government of South Georgia and the South Sandwich Islands.

### Device deployment and retrieval

**FIGURE 1:**
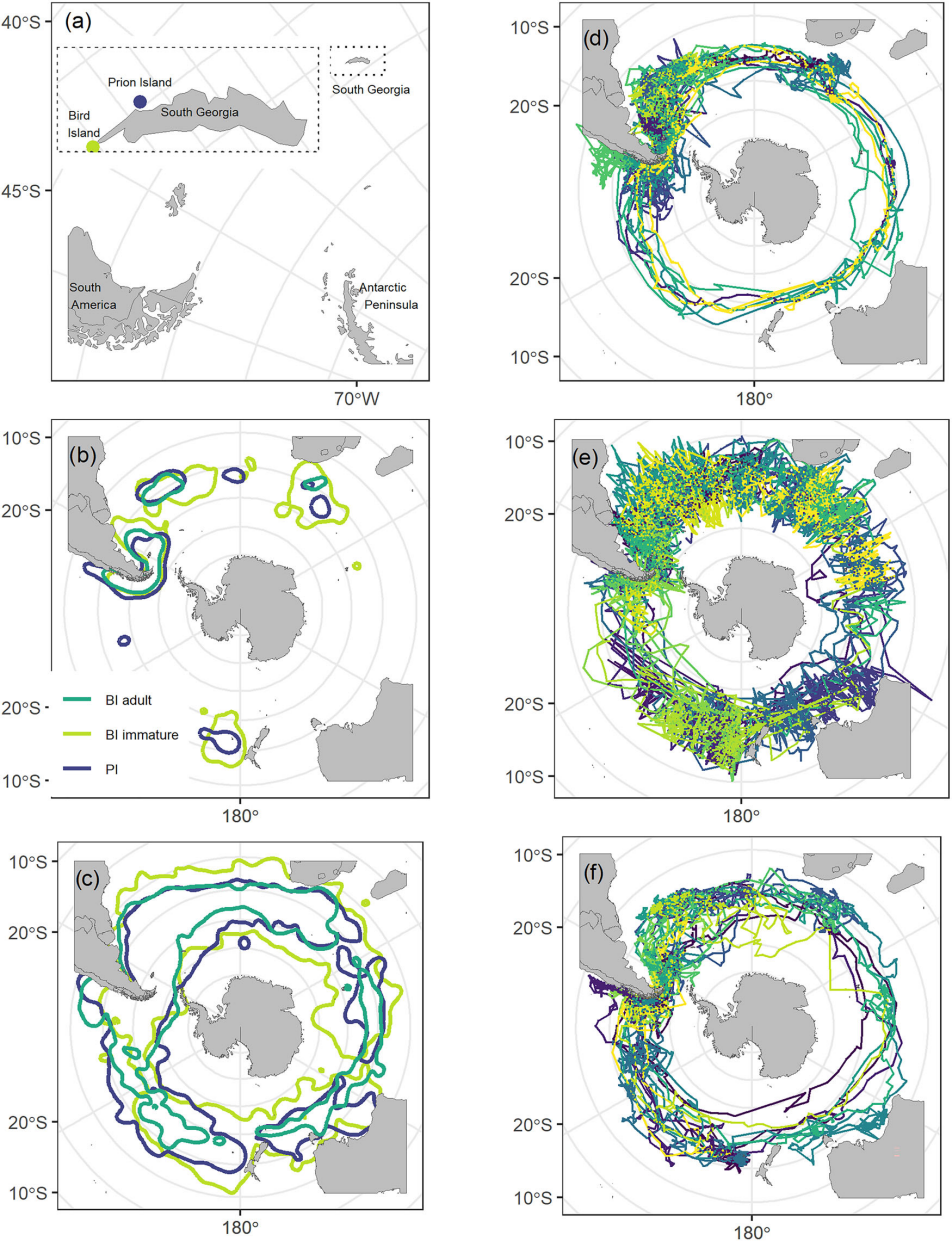
Locations of (a) Bird Island (BI) and Prion Island (PI), South Georgia, where nonbreeding wandering albatrosses were tracked with geolocators; albatross (b) core areas (50% utilization distribution), (c) home ranges (95% utilization distribution), and tracks from (d) Bird Island in 2022 (adults) and (e) 2006 (immatures) and (f) Prion Island in 2022 (older immatures and possibly some nonbreeding adults).

Wandering albatrosses were tracked from two colonies at South Georgia (Figure 1) between January and December 2022. Bird Island (54°00ʹ S, 38°03ʹ W) and Prion Island (54°02ʹ S, 37°25ʹ W) are located ∼50 km apart and held ∼770 and ∼37 breeding pairs, respectively, in austral summer 2014–2015 (Poncet et al., 2017). At Prion Island, geolocators (Global Location Sensors or GLS loggers; Intigeo C330, Migrate Technology) were deployed on 20 nonbreeding birds in January 2022; 14 were recovered in December 2022, 12 of which provided data. Although these were unringed and of unknown breeding history, based on plumage (largely white) and early colony attendance (January) in both seasons, these were likely to be mainly older immatures and potentially some nonbreeding adults (i.e., deferring breeders) (Pickering, 1989; Tickell, 1968). At Bird Island, 12 geolocators deployed on breeding adults in January 2021 were recovered in January 2023. All active nests at Bird Island are checked at least monthly, and so we know that these bred successfully in 2021 and were not breeding in January 2022, consistent with the individuals tracked from Prion Island. The geolocators were fixed by cable ties to plastic rings deployed on the leg and took <2 min to deploy and retrieve. They were set to mode 9; light was sampled every minute, and the maximum was recorded after 5 min. Temperature was measured every 8 h after continuous immersion for 20 min.

Given that distributions of wandering albatrosses vary with age and breeding status (Clay et al., 2018; Weimerskirch et al., 2014), and the histories of birds from Prion Island were unknown, we also compared their distributions with 18 immature wandering albatrosses tracked from Bird Island in January to December 2006 (Clay et al., 2019), in order to disentangle the effect of breeding site from that of age. Nonbreeding wandering albatross migration strategies and foraging zones were highly consistent from year to year (Weimerskirch et al., 2015); hence, we assumed that the data from 2006 were representative of birds of similar status in later years. As at Prion Island, the immatures tracked at Bird Island departed the colony between December and March and returned between October and December.

### Data processing

Light data collected in 2022 were processed using ProbGLS (Merkel et al., 2016). This uses light data to create a cloud of points for each timestamp, which is then refined using sea surface temperature (SST) and travel speed to weight each point and identify the most likely track. Timestamps were weighted as zero where SST was not recorded or differed by >3°C from contemporaneous remotely sensed SST data, or where speeds were unrealistic (70 or 5 m/s for periods in flight or on the water, respectively). Nonzero weighted data were linearly interpolated to provide two positions per day for each individual. A benefit of this approach was that it was usually not necessary to exclude points around the equinoxes, as the latitude was constrained by SST. However, for one individual, locations collected during the equinox seemed unlikely (very close to the Antarctic continent) and were removed from the analyses. The data from 2006 had already been processed following the methods described in Clay et al. (2019). Although different approaches may affect location estimates, particularly latitudes (Merkel et al., 2016), this was highly unlikely to have a material effect on our conclusions.

To avoid biases associated with the time spent attending the colony, we only compared distributions of nonbreeding birds when they were not acting as central‐place foragers by including data from the date in December to March that each individual left a 500‐km buffer around the colony for over 30 days, until it returned to this buffer area in the following October to December. A bootstrapping approach was undertaken to assess the representativeness of the samples for each group with the R package Track2KBA (Beal, Oppel, et al., 2021). All analyses were conducted in R 4.3.2 (R Core Team, 2021).

### Spatial segregation

To test for differences in spatial distribution by colony or status, utilization distributions (UDs) representing the core area (50% UD) and home range (95% UD) were calculated for each of the three groups and for each individual. The smoothing parameter (200 km) and the grid cell size (50 km) were consistent across sites and account for uncertainty in location estimates from geolocator data (Phillips et al., 2004). Kernel analysis and spatial overlap were run in track2KBA.

Pairwise population‐level overlaps in UDs were calculated for the three groups with Bhattacharya's affinity (BA), which is a metric of similarity between two distributions, and a randomization procedure was used to identify significant differences following Clay et al. (2018). First, overlaps among the groups of tracked birds were calculated. Subsequently, each track was randomly assigned to a group, and new UD and BA were calculated. This was repeated 1000 times, and the proportion of occurrences where the randomly assigned overlap was smaller than our sample overlap provided the *p* value. The proportions of time spent in different sectors of the Southern Ocean were calculated for each individual. The sizes of the core area and home range were compared using linear models and post hoc Tukey tests.

### Overlap with fisheries

Data on monthly fishing activity (hours fished) were downloaded from Global Fishing Watch (Global Fishing Watch, 2025) at 1° resolution. Spatiotemporal overlap between the tracked birds and fishing was calculated for drifting (pelagic) and set (demersal) longlines, trawlers, squid jiggers, unidentified fishing, and all fishing combined, for each flag state. The proportions of time spent by birds from each group in each 1° grid cell each month were calculated and multiplied by the number of hours fished in that cell to provide a monthly overlap index. The same approach was also used to calculate an overlap index for birds according to their migration strategy (as opposed to breeding site and age).

## RESULTS

Tracks were obtained for 12 nonbreeding adult wandering albatrosses and 18 immatures at Bird Island in 2022 and 2006, respectively, and 12 older immatures or nonbreeding adults at Prion Island in 2022. Date of return to the colony varied little among groups: almost all individuals returned in the last 2 weeks of November or the first week of December. Date of departure was much more variable; nonbreeding adults from Bird Island all departed between early December and late January, whereas immature birds from Bird Island and birds from Prion Island departed between mid‐January and late March. The core areas were well represented for all three groups (Bird Island adults, 92%; Bird Island immatures, 91%; Prion Island, 89%), whereas the home range was slightly less representative (Bird Island adults, 83%; Bird Island immatures, 84%; Prion Island, 82%) but sufficient to make population‐level inferences about distributions. These values represented the percentage of the area used by the population that was captured in our sample.

### Spatial segregation

**FIGURE 2:**
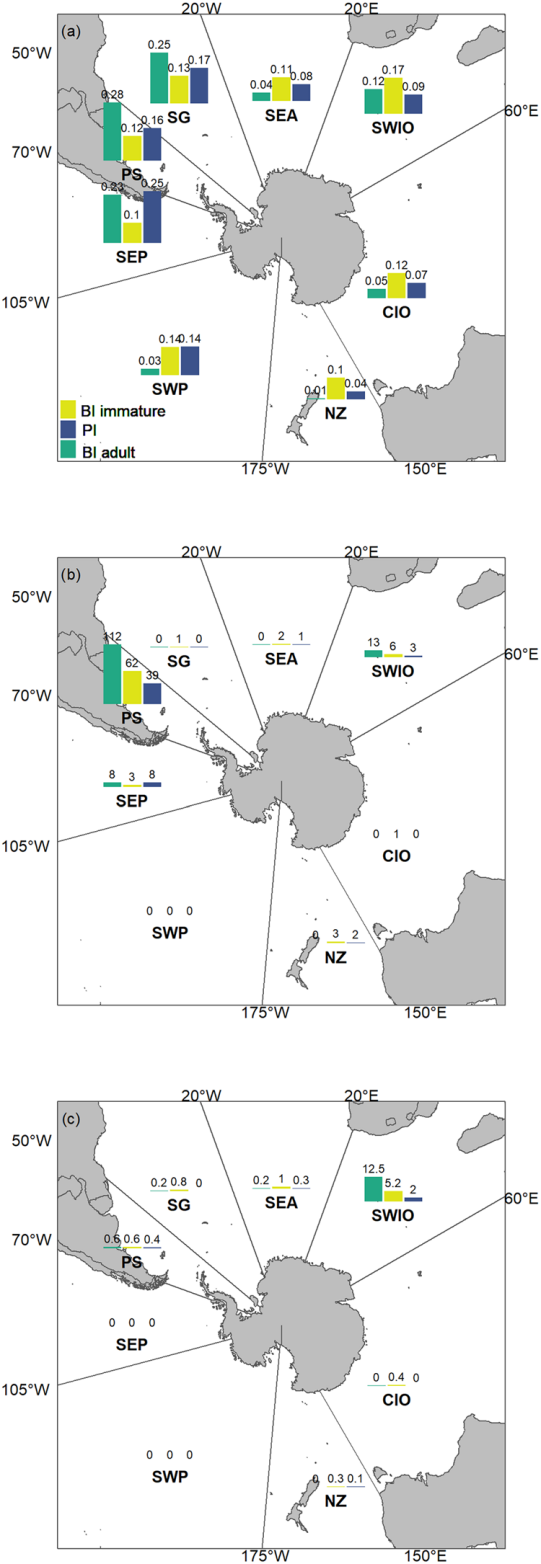
(a) The proportion of time spent in different ocean sectors by nonbreeding wandering albatrosses from Prion Island (PI) and Bird Island (BI), (b) index values of overlap of nonbreeding wandering albatrosses with all fishing activity, and (c) index values of overlap of nonbreeding wandering albatrosses with drifting (pelagic) longline fishing (PS, Patagonian Shelf; SG, South Georgia; SEA, southeast Atlantic Ocean; SWIO, southwest Indian Ocean; CIO, central Indian Ocean and Australia; NZ, New Zealand; SWP, southwest Pacific Ocean; SEP, southeast Pacific Ocean).

Some individuals from all three groups circumnavigated Antarctica, whereas others remained in the southwest Atlantic and Patagonian Shelf area (Figure 1; Appendices S1–S3). The core areas of all three groups were on the Patagonian Shelf, southwest Atlantic Ocean, southwest Indian Ocean, and the Chatham Rise (birds from Prion Island, and immatures but not nonbreeding adults from Bird Island). These varied more among birds from Prion Island and immatures from Bird Island than nonbreeding adults from Bird Island (Appendix S4). Immatures from Bird Island had larger foraging areas than individuals from Prion Island and nonbreeding adults from Bird Island (*F*_39_ = 12.97, *p* < 0.01). The home range for all three groups was circumpolar and of similar mean size (Figure 1). UDs of immatures and adults from Bird Island showed significant spatial segregation in both the core foraging area and the home range area (Table 1). Spatial segregation was also detected in the home range of birds from Prion Island and immatures from Bird Island (Table 1). The home range of immatures from Bird Island was evenly distributed around Antarctica, whereas that of birds from Prion Island was more focused on the southwest Atlantic and Pacific than the Indian Ocean. In contrast, wandering albatrosses from Prion Island and immatures from Bird Island spent more time in ocean basins farther from South Georgia than adults from Bird Island; this last group spent a higher proportion of time on the Patagonian Shelf and around South Georgia (Figure 2). In terms of time spent in different ocean sectors, the distribution of birds from Prion Island was intermediate between those of immatures and adults from Bird Island.

**TABLE 1:** Observed and randomized overlap (Bhattacharya's affinity) of utilization distributions of nonbreeding wandering albatrosses tracked from Bird Island (BI) and Prion Island (PI), South Georgia.

Population	Utilization distribution (%)^a^	Sample overlap	Mean randomized overlap (SD)	*p* ^b^
BI adults–Prion Island	50 (core area)	0.34	0.36 (0.04)	0.35
	95 (home range)	0.77	0.78 (0.03)	0.41
BI immatures–BI adults	50 (core area)	0.28	0.37 (0.03)	0.01
	95 (home range)	0.66	0.80 (0.03)	<0.001
BI immatures–Prion Island	50 (core area)	0.30	0.37 (0.03)	0.22
	95 (home range)	0.74	0.80 (0.03)	0.03

^a^ Maximum values for the core area are 0.5 and for the home range area are 0.95.

^b^ Proportion of randomized overlaps smaller than the observed overlap.

The majority of individuals adopted one of four migration strategies: resident, birds remained within a few thousand kilometers of South Georgia and frequented the Patagonian Shelf and Humbolt Current region; to South Africa and farther east, individuals flew east with prevailing winds and used stopover sites around the Subantarctic Front (SAF) off South Africa or farther east toward Australia; circumnavigation, individuals flew east with prevailing winds and circumnavigated Antarctica at least once, sometimes including stopovers in the SAF off New Zealand, Australia, or South Africa; and west to New Zealand, individuals flew west against prevailing winds in the Pacific Ocean, spending time in the Chatham Rise. Each of the four migration strategies was adopted by at least two individuals from each group, except that no nonbreeding adults from Bird Island traveled west across the Pacific Ocean (Appendix S5).

### Overlap with fisheries

**FIGURE 3:**
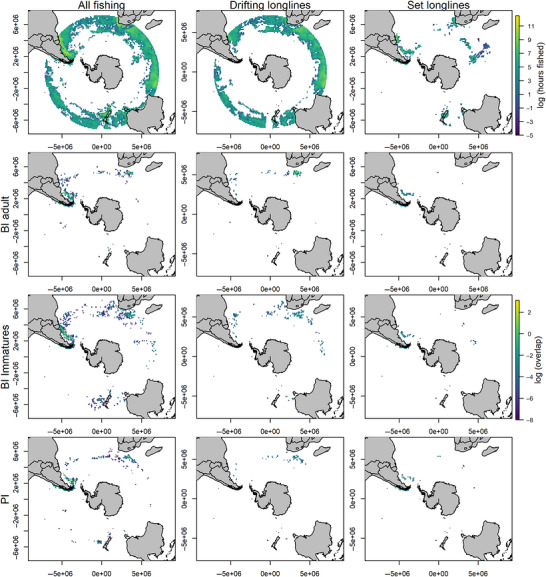
All fishing activity, drifting (pelagic) longline, and set (demersal) longline in 2022 and their overlap with nonbreeding wandering albatrosses tracked from Bird Island (BI) (adults in 2022, immatures in 2006) and Prion Island (PI) in 2022. The monthly overlap is summed to produce annual overlap maps.

**FIGURE 4:**
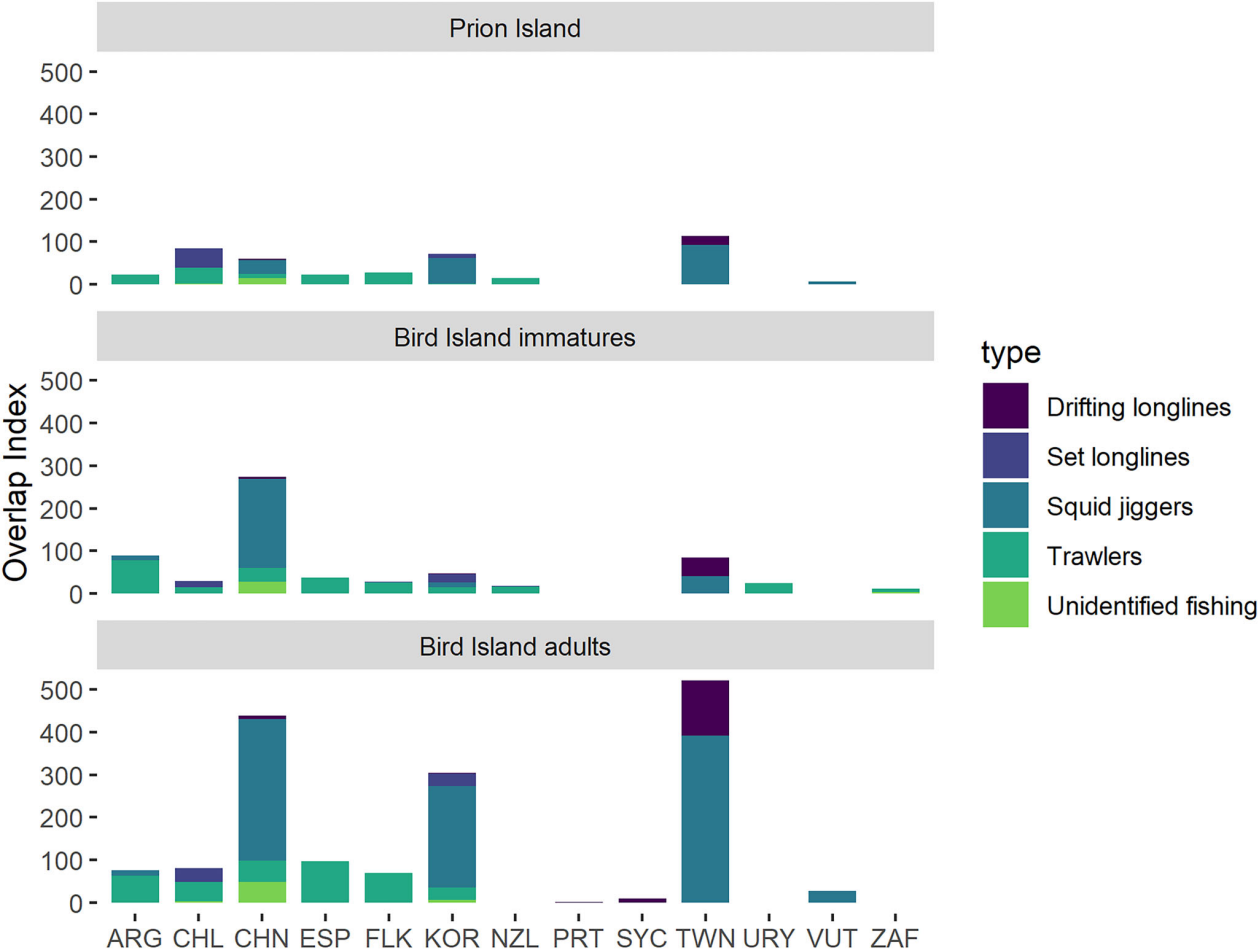
Overlap of nonbreeding wandering albatrosses from Bird Island and Prion Island, South Georgia, with fishing vessels by flag state and gear type (fishing gears, primary type for vessels actively fishing; three‐letter codes, registered flag state; ARG, Argentina; CHL, Chile; CHN, China; ESP, Spain; FLK, Falkland Islands; KOR, South Korea; NZL, New Zealand; PRT, Portugal; SYC, Seychelles; TWN, Taiwan; URY, Uruguay; VUT, Vanuatu; ZAF, South Africa).

Fishing activity occurred throughout the circumpolar range of the birds from all groups (Figure 3). Overlap with fishing of nonbreeding adults from Bird Island was more than three times higher than for individuals from Prion Island and more than twice that of immatures from Bird Island. Overlap with drifting (pelagic) longlines and squid jiggers by nonbreeding adults from Bird Island was more than five times greater than for birds from Prion Island and twice that of immatures from Bird Island (Table 2). Birds from all groups overlapped with multiple fishing fleets on the Patagonian Shelf, the southwest Atlantic Ocean, and around South Africa, particularly between March and June (Table 2; Figure 3). Birds from Prion Island and immatures from Bird Island also overlapped with trawlers off New Zealand. Overlap was highest with Chinese, South Korean, and Taiwanese squid jiggers, Taiwanese drifting (pelagic) longlines, and Argentinian and Spanish trawlers (Figure 4). Overlap with fishing was considerably lower for birds that travelled east to New Zealand than for birds that adopted any other migratory strategy (Appendices S6 & S7). Overlap with all fishing types was similar for the remaining three strategies, except that birds that traveled east of South Africa had a higher overlap with drifting (pelagic) longlines than birds from other groups.

**TABLE 2:** Index of the overlap between fisheries and nonbreeding wandering albatrosses tracked from Bird Island (BI) in 2006 (immatures [im]) and 2022 (adults [ad]) and Prion Island (PI), South Georgia, in 2022.

	All fishing			Drifting (pelagic) longlines			Set (demersal) longlines			Trawlers			Squid jiggers			Unidentified fishing		
Month	BI ad	PI	BI im	BI ad	PI	BI im	BI ad	PI	BI im	BI ad	PI	BI im	BI ad	PI	BI im	BI ad	PI	BI im
January	149	0	6	3	0	0	5	0	6	28	0	0	95	0	0	17	0	1
February	97	12	17	0	0	1	14	3	7	27	7	8	45	1	0	5	0	3
March	491	33	87	1	0	2	11	9	6	107	16	38	353	6	38	12	2	6
April	525	168	138	20	0	9	11	11	6	104	47	28	365	107	87	18	1	20
May	255	159	245	15	11	20	12	17	4	65	31	71	146	81	126	13	14	5
June	78	42	129	50	5	17	5	5	6	13	18	73	3	2	28	6	6	4
July	20	16	41	16	0	7	1	2	2	0	11	25	0	0	0	0	1	1
August	48	29	24	44	17	15	1	4	2	2	7	4	0	0	0	0	2	0
September	20	11	8	12	0	1	0	1	0	8	8	7	0	0	0	1	0	1
October	9	16	24	1	0	2	3	8	6	5	7	15	0	0	0	0	0	0
November	8	6	6	1	0	1	5	4	3	1	1	2	0	0	0	1	0	1
December	19	1	0	0	0	0	8	1	0	10	7	0	1	0	0	17	0	0
Total	1719	493	725	163	33	75	76	65	48	370	160	271	1008	197	279	90	26	42

## DISCUSSION

This study is one of the first to link spatial segregation, relative fisheries overlap, and hence likely bycatch risk of nonbreeding albatrosses or petrels to differences in population trends among breeding sites in the same island group. There was extensive individual variation in the distribution of nonbreeding wandering albatrosses from each site. Differences in distribution between the three groups of birds that we tracked were more apparent in the proportion of time spent in different ocean sectors than in the overall core areas and home ranges; these were most obvious between age classes rather than colonies. Regardless, both nonbreeding adults and immatures from Bird Island overlapped more with fisheries than birds (mostly older immatures) from Prion Island. This has important implications for bycatch risk, which is by far the greatest threat to wandering albatrosses at South Georgia (Mackley et al., 2025; Phillips et al., 2016).

### Distribution and spatial segregation

Wandering albatrosses from both colonies used one of four migratory strategies. Three of these were to remain resident, migrate east to South Africa or Australia, or circumnavigate Antarctica, which were the strategies adopted by wandering albatrosses breeding in the southern Indian Ocean and by gray‐headed albatrosses (*Thalassarche chrysostoma*) from South Georgia (Croxall et al., 2005; Weimerskirch et al., 2015). However, birds from Prion Island and immatures from Bird Island also used a fourth strategy, migrating west into prevailing winds as far as New Zealand. This is contrary to the general pattern that wandering albatrosses travel predominantly with prevailing winds, to such a degree that they may detour many thousands of kilometers in the nonbreeding period in order to take advantage of wind regimes (Weimerskirch et al., 2000, 2015). However, seabirds can use low‐pressure systems to fly westwards in regions predominantly influenced by westerly winds (Murray et al., 2003). All four individuals that adopted this strategy departed from South Georgia between late February and late March, and it is plausible that the pressure systems at this time of year facilitated this westerly migration.

The spatial segregation of immature and nonbreeding adult wandering albatrosses has likely evolved to reduce competition for foraging opportunities around the large breeding colonies at South Georgia and in the Indian Ocean. Juvenile and immature seabirds frequently avoid competition with adults by spending more time in lower‐quality habitats (Clay et al., 2016; Phillips et al., 2017; Weimerskirch et al., 2006). In contrast, nonbreeding adults can presumably compete more successfully with breeding birds near colonies, and high marine productivity in the southwest Atlantic may be sufficient to support large numbers of wandering albatrosses year‐round (Croxall & Wood, 2002; Wakefield et al., 2014), particularly given that density‐dependent competition has likely reduced along with the steep population decline (by 39% between 1983/84 and 2023/24; Mackley et al., 2025). This would explain the higher proportion of time spent by nonbreeding adults around South Georgia and on the Patagonian Shelf. Additionally, lack of prior knowledge of productive foraging habitats, combined with differential responses to external environmental cues, may drive young individuals to disperse more widely, whereas experienced adults migrate directly to productive locations (Frankish et al., 2020). It is plausible that some of the differences in UDs, particularly in terms of latitude, may reflect the different geolocator processing methods (Merkel et al., 2016). However, the processing would have minimal impact on the proportion of time spent in different ocean sectors, and so we are confident that our results represent true differences in distributions between age classes and sites.

Sexual segregation as a result of size dimorphism, competitive exclusion, or reproductive role also mediates the distribution of seabirds during the nonbreeding season (Clay et al., 2018; Pérez et al., 2014; Phillips et al., 2009; Weimerskirch et al., 2015). Sex was unknown for the majority of individuals in this study, and thus, we cannot rule out an effect. However, for nonbreeding adults tracked from Bird Island, both males and females adopted three of the four migration strategies, suggesting that sex was not a major driver of the observed variability in distribution.

Spatial segregation between conspecific seabirds from different island groups during the nonbreeding season is often considered to be a response to habitat or prey specialization, variability in breeding strategy, or niche partitioning (Clay et al., 2016; Thiebot et al., 2012; Weimerskirch et al., 2015). It was not possible in our study to fully disentangle the effects of breeding site from those of age, because although the majority of birds from Prion Island were older immatures, the sample possibly also included a few nonbreeding adults. However, there was clearly a site effect given the spatial segregation in home ranges between these birds and immatures from Bird Island, with more time spent by the former in the southeast Pacific and less time around New Zealand. The reason for these differences is unclear. Wandering albatrosses breeding at Bird Island and Prion Island show spatial segregation but use habitats with similar characteristics, providing no evidence for consistent differences in habitat specialization (Warwick‐Evans et al., 2026). Wandering albatrosses at Bird Island breed biannually if successful (Tickell, 1968), and there is no reason to consider that breeding strategies differ at Prion Island. Niche partitioning between the two populations is a possibility, but this seems unlikely given the large overlap and similar migration strategies of birds from all three groups. Just 50 km separates Bird Island from Prion Island, which is just a fraction of the vast distances travelled by nonbreeding wandering albatrosses. There are no clear ecological or physiological reasons why the birds from the two islands should segregate when there is no central‐place foraging constraint. The ecological drivers mediating the remarkable spatial segregation observed between these two populations during the breeding season were similarly unclear (Warwick‐Evans et al., 2026). Instead, the segregation of breeding and nonbreeding birds may be driven by a combination of past experience, information exchange, and cultural evolution. There is no evidence that migration strategy is heritable in wandering albatrosses (Weimerskirch et al., 2015). Instead, individual experience as a juvenile or immature influences migration patterns and distributions of seabirds in later life, which are often refined over time (Campioni et al., 2020; Guilford et al., 2011; Votier et al., 2017).

### Overlap with fisheries

Although spatial segregation between age classes and breeding colonies for the nonbreeding wandering albatrosses was subtle, there were striking contrasts in the overlap with fisheries. If the latter reflects relative bycatch risk, that would at least partly explain the contrasting rates of population decline at Bird Island and Prion Island. Although it has been suggested previously that spatial segregation between populations on different island groups may affect bycatch risk of nonbreeding adults (Clay et al., 2016; Weimerskirch et al., 2015) or breeders (Corbeau et al., 2021; Genovart et al., 2018), ours is the first study to quantify this for colonies within the same island group and to show it for immature birds.

Wandering albatrosses are attracted to many types of fishing vessel including squid jiggers, trawlers, and longliners (Carneiro, Clark, et al., 2022); however, pelagic (drifting) and demersal (set) longline fisheries are thought to pose the greatest threat in the absence of bycatch mitigation, as birds attempting to feed on the baited hooks are caught and drowned (Jiménez et al., 2014, 2016). We also analyzed overlaps with squid jiggers and trawlers, as some mortalities of wandering albatrosses have been recorded (Adasme et al., 2019; FIG, 2021; Reid et al., 2021), but these seem unlikely to represent as great a threat as other fishing modalities. Overlap of nonbreeding adults from Bird Island with all fishing, and with drifting (pelagic) longlines, was considerably higher than that of both immatures from Bird Island and birds from Prion Island. Bycatch of wandering albatrosses in pelagic longline fisheries in the southwest Atlantic is higher for adults than juveniles or immatures (Croxall & Prince, 1990; Jiménez et al., 2016). This pattern for seabirds may result from the competitive advantage of adults behind vessels but is more commonly a consequence of spatial segregation (Clay et al., 2019; Collet et al., 2017; Frankish et al., 2021; Gianuca et al., 2019). Although the distribution and use of ocean sectors of birds from Prion Island were intermediate between those of adult and immature birds from Bird Island, their overlap with fisheries was usually the lowest of all three groups. This suggests that relatively fine‐scale variation in spatial or temporal distribution can have a large effect on fisheries overlap metrics. Indeed, although there are hotspots of fishing effort within the circumpolar range of seabirds from all three groups that we tracked, temporal overlap was lower in the more distant regions, in which immature birds from Bird Island and birds from Prion Island spent more time. There were also some differences between these two groups, as the former overlapped more with fisheries around New Zealand and off South Africa.

Understanding migration strategies at the individual level may provide valuable insights when evaluating the overlap between seabirds and fisheries. The wandering albatrosses that remained resident, migrated east past South Africa, or circumnavigated Antarctica tended to spend time in regions of high fishing activity in the southwest Atlantic or Subantarctic Front off South Africa (Clay et al., 2019, this study). In contrast, birds that travelled west to New Zealand spent little time in these areas, and fishing activity between South America and New Zealand tends to occur at lower latitudes, resulting in lower fisheries overlap.

For all groups of birds, overlap with fishing activity was largely concentrated on the Patagonian Shelf and shelf break, whereas overlap with drifting (pelagic) longline activity was concentrated at the Subantarctic Front in the southwest Indian Ocean, consistent with a previous study (Clay et al., 2019). This explains why the wandering albatrosses that migrated east past South Africa had a higher overlap with drifting (pelagic) longlines than those with alternative migration strategies. Bycatch of wandering albatrosses in subtropical tuna fisheries around the Brazil–Falklands Confluence is high (Bugoni et al., 2008; Jiménez et al., 2014) and likely a major cause of the long‐term population decline at South Georgia (Pardo et al., 2017). Bycatch rates were also high in pelagic longline fisheries in the southwest Pacific (Gales et al., 1998; Huang & Yeh, 2011). Although overlap with pelagic and demersal fisheries operating off South Africa and in the southwest Indian Ocean is high, the available data from observers suggest that bycatch rates for wandering albatrosses in these regions are low (Jiménez et al., 2020; Petersen et al., 2009; Rollinson et al., 2017; Ryan et al., 2002). Additionally, breeding populations of wandering albatrosses in the Indian Ocean are stable (Weimerskirch et al., 2018), suggesting that bycatch of this species is much less of a problem in this region than elsewhere. This may reflect differences in vessel operations (e.g., time of setting, distance between weight and hook, and heaviness of weight), the use of mitigation, or other factors, such as the assemblage of species scavenging behind vessels. The white‐chinned petrel (*Procellaria aequinoctialis*) is the most bycaught seabird species in the Southern Ocean (Anderson et al., 2011). They bring baited hooks back to surface waters but are often displaced by larger seabirds, such as albatrosses, putting the latter at risk (Jiménez et al., 2012). Globally, the highest abundance of white‐chinned petrels is in the southwest Atlantic, which is likely a key factor increasing the bycatch of seabirds in general in this region and means there is not a linear relationship between overlap or attendance rates and the probability of capture for different vessel types or locations (Carneiro, Clark, et al., 2022).

An important caveat is that the overlap metrics approximate absolute risk because impacts also depend on bird behavior and operational factors, including vessel attraction, gear type, or the use of bycatch mitigation measures (Carneiro, Clark, et al., 2022; Collet et al., 2017; Phillips et al., 2016). The use of one or more of bird‐scaring (streamer or tori) lines, night setting, or heavier line weighting by demersal and pelagic longline vessels can reduce seabird bycatch (Collins et al., 2021; Da Rocha et al., 2021; Jiménez et al., 2020). Although bycatch reduction is greatest when these are used in combination, mitigation requirements for many fishing fleets are still not best practice, and monitoring of compliance is insufficient (Phillips et al., 2016). Indeed, requirements for bycatch mitigation and observer coverage differ greatly among the regional fisheries management organizations that regulate pelagic or demersal fishing in each region (Appendix S8). Accordingly, the relationship between our fisheries overlap index and bycatch risk will vary according to the fleets or vessels involved.

Overlap was highest with squid jiggers flagged to Taiwan, China, or South Korea, followed by trawlers flagged to Argentina, Spain, and the Falkland Islands for birds for all groups. Moreover, birds from all three groups overlapped with drifting (pelagic) longlines from Taiwan and China and set (demersal) longlines from Chile and South Korea. In all cases, overlap was higher for adults from Bird Island than for immatures from Bird Island or birds from Prion Island. Although the overlap with squid jiggers and trawlers was greater in terms of fishing hours, bycatch risk is considerably higher in demersal and pelagic longline fisheries even though the encounters are less frequent and of shorter duration (Carneiro, Clark, et al., 2022; Clay et al., 2019). Differences in bycatch rate also vary according to flag state as a result of different operational or mitigation measures, and bycatch risk may also be underreported or biased by low observer coverage (Clay et al., 2019; Phillips, 2013).

Another caveat is that the GFW database does not provide a complete picture of fishing effort. The system relies on the automatic identification system (AIS) to identify vessels and an algorithm to detect fishing activity from vessel movement patterns (Global Fishing Watch, 2025). However, many small fleets operating in coastal waters, and some larger fleets on the high seas, including illegal, unreported, and unregulated vessels, do not have AIS or it has been disabled, and in some regions, the satellite coverage is poor (Carneiro, Dias, et al., 2022; Orben et al., 2021). Additionally, the algorithm that detects fishing activity is not always correct (Kroodsma et al., 2019). However, for the purposes of our study, it is likely that biases are similar for birds from each group, and thus any impact on our conclusions will be minimal.

### Implications for population trajectories and management

Our study shows considerably higher overlap with fisheries of nonbreeding wandering albatrosses from Bird Island than from Prion Island, and it is likely this contributes to the contrasting rates of decline (Mackley et al., 2025; Rackete et al., 2021). Overlap of nonbreeding adults was higher than that of younger birds, and in long‐lived seabirds like albatrosses, increased adult mortality will have a greater impact on the population trends than mortality of immatures, given the slow rate of reproduction (Lewison et al., 2014). Despite the low proportion of overlap of wandering albatrosses from all three groups with longline fishing on the Patagonian Shelf and at the Brazil–Falklands Confluence, the overlap index in this region was higher for birds from Bird Island than from Prion Island, consistent with the contrasting population declines. There are no threats on land in South Georgia, and wandering albatrosses are highly philopatric (Gauthier et al., 2010). As such, bycatch in fisheries is the only plausible explanation for the population decline (Pardo et al., 2017). Higher rates of overlap with fishing activity in the southwest Atlantic during the breeding season indicate that bycatch risk for wandering albatrosses is greatest when adults are most constrained (Warwick‐Evans et al., 2026). Consequently, bycatch in fisheries of breeding birds most likely contributes more than that of nonbreeding birds to the declines.

Our study highlights variability in spatial distribution, and consequently fisheries overlap, in relation to life‐history stage and breeding site, underlining the importance of considering these factors in seabird–fisheries risk assessments and when making management decisions (Carneiro et al., 2020; Small et al., 2013). By improving our knowledge of the spatial and temporal overlap of seabirds with fisheries, mitigation requirements and monitoring of compliance can be targeted at relevant fishing fleets to reduce the threat to these iconic and threatened seabirds.

## AUTHOR CONTRIBUTIONS

R.A.P. and V.W‐E designed the project and planned the fieldwork. V.W‐E and E.J.P. carried out the fieldwork. V.W‐E did the analysis and wrote the original draft. All authors reviewed and edited the text.

## Supporting information



Supporting Information

## Data Availability

All tracking data are available from the seabird tracking database (https://www.seabirdtracking.org/)
