# Supplementary material for: Overlap of nonbreeding wandering albatrosses with fisheries and implications for colony‐specific population trajectories at South Georgia
Source: Conserv Biol. 2026 Mar 23;40(4):e70260. doi: 10.1111/cobi.70260 (PMC13392789; doi:10.1111/cobi.70260)
Supplement: Supplementary file 1 — Supporting Information [file COBI-40-e70260-s001.docx]

**Could relative fisheries overlap of non-breeding wandering albatrosses explain colony-specific population trajectories at South Georgia?**

Supplementary Materials

Appendix S1: Migration tracks from non-breeding wandering albatrosses tagged with geolocators at Prion Island, South Georgia


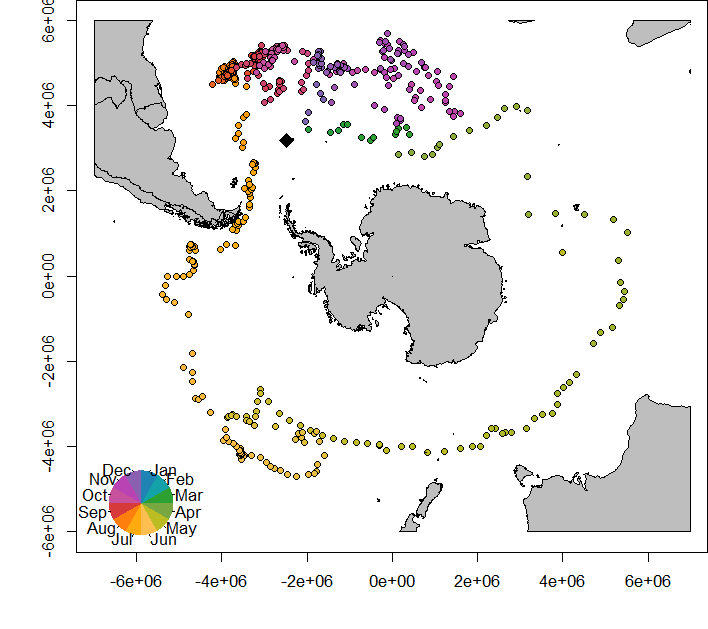

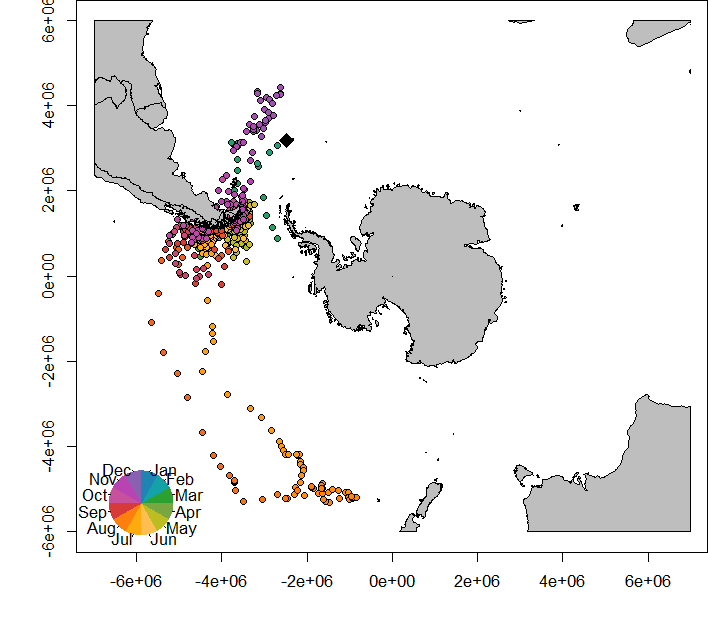

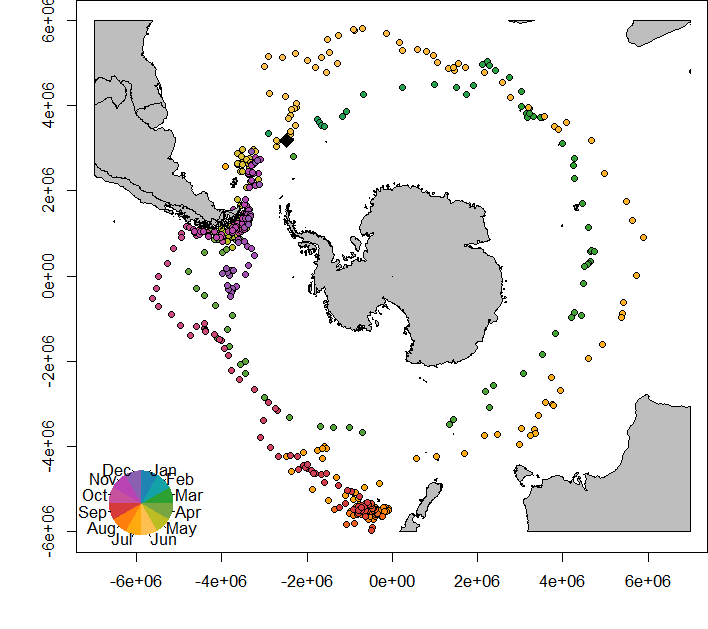

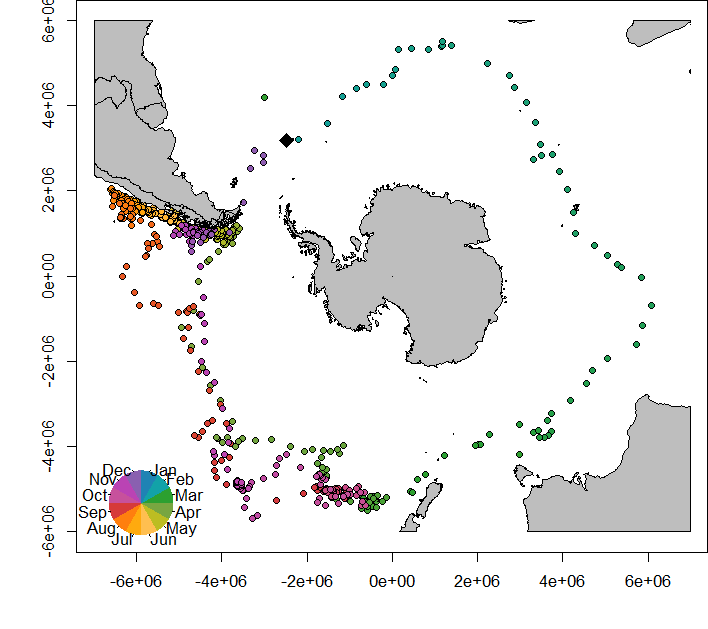

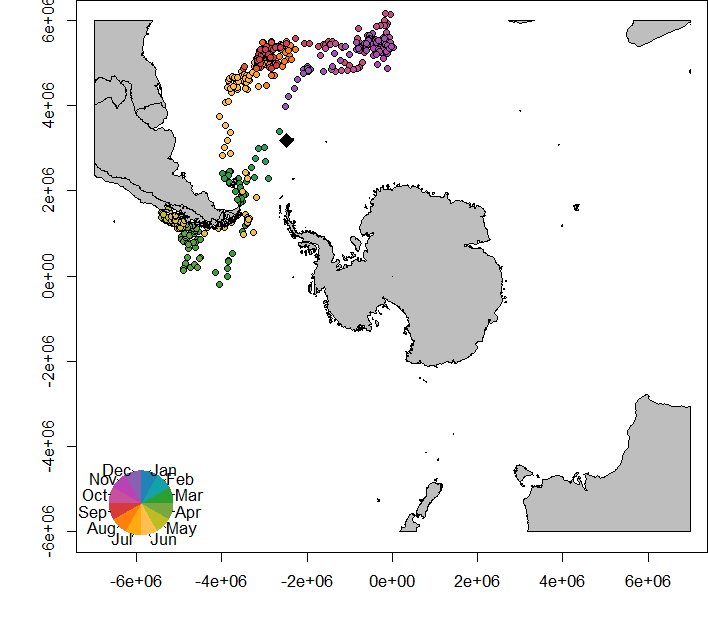

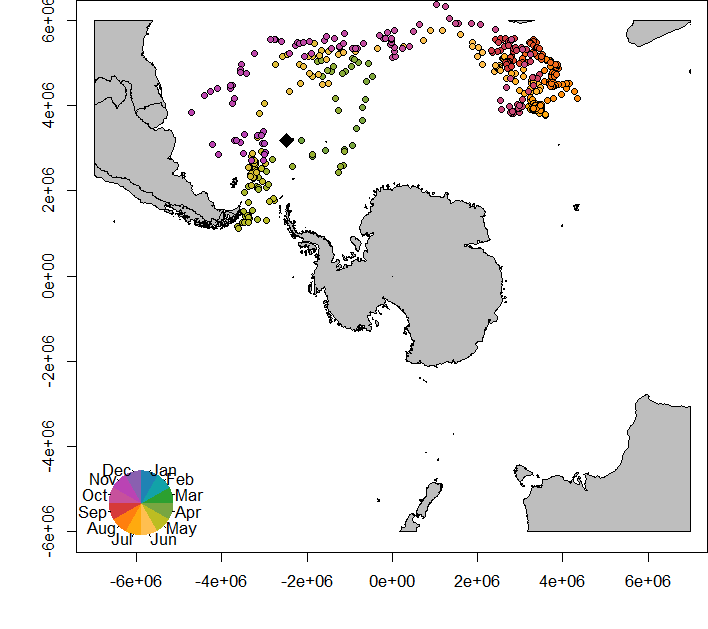

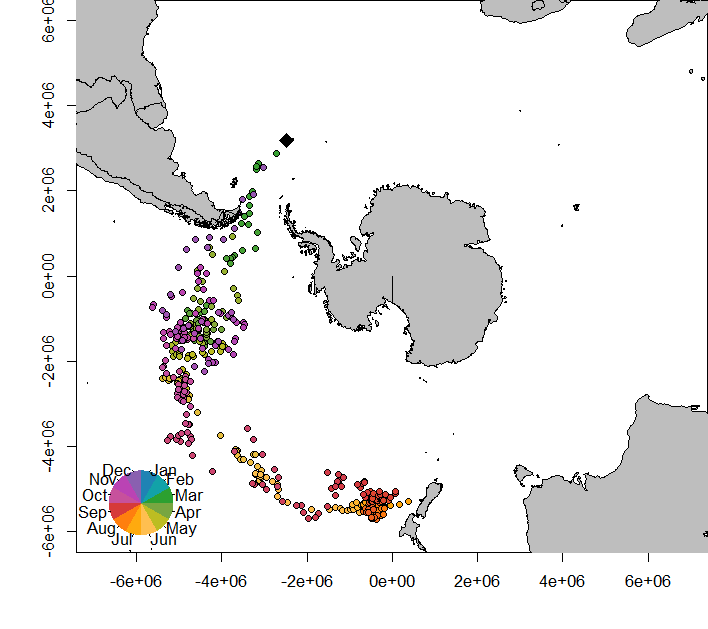

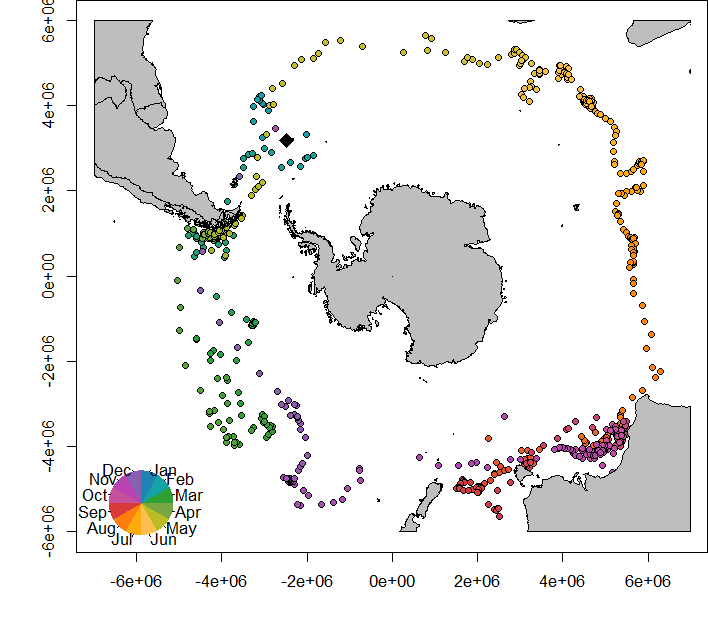

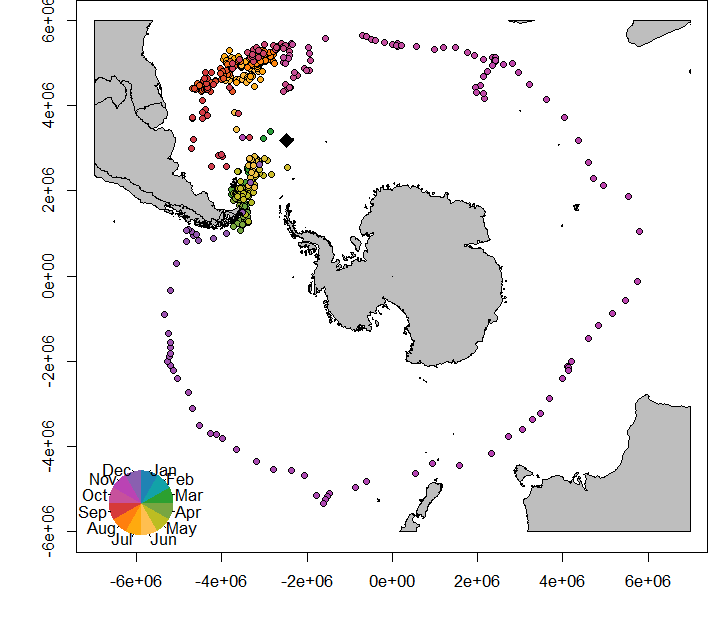

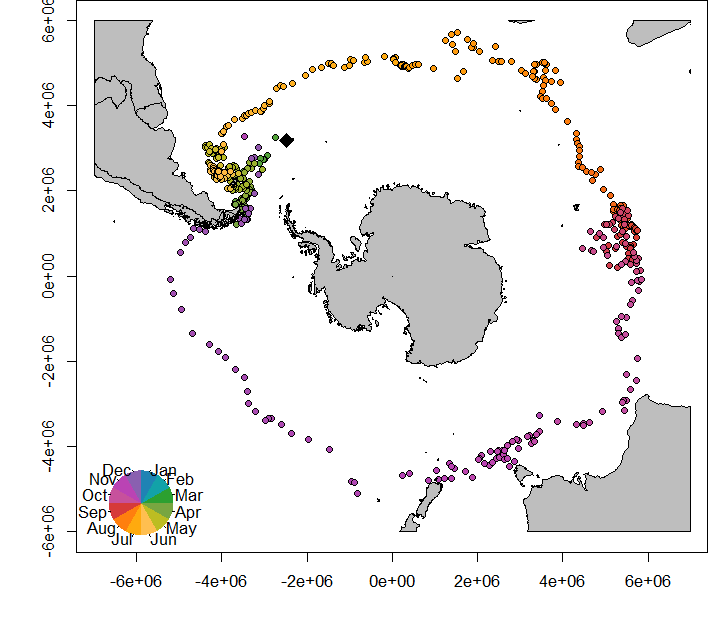

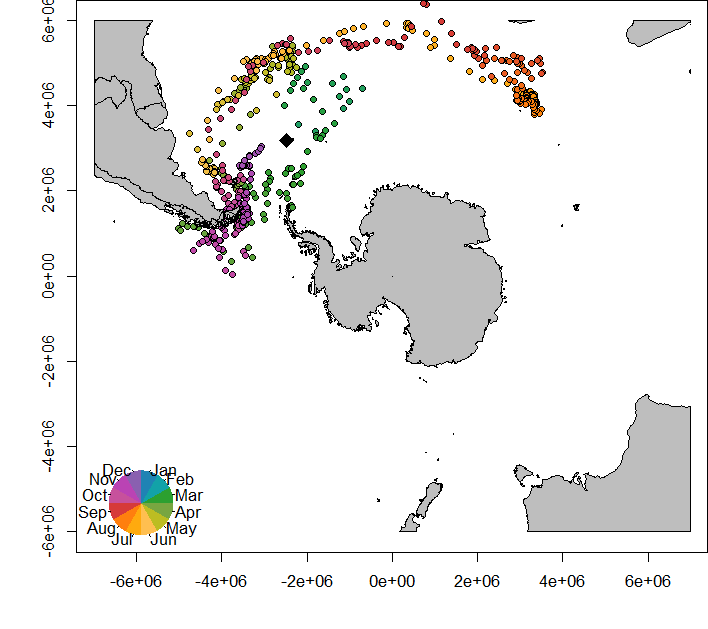

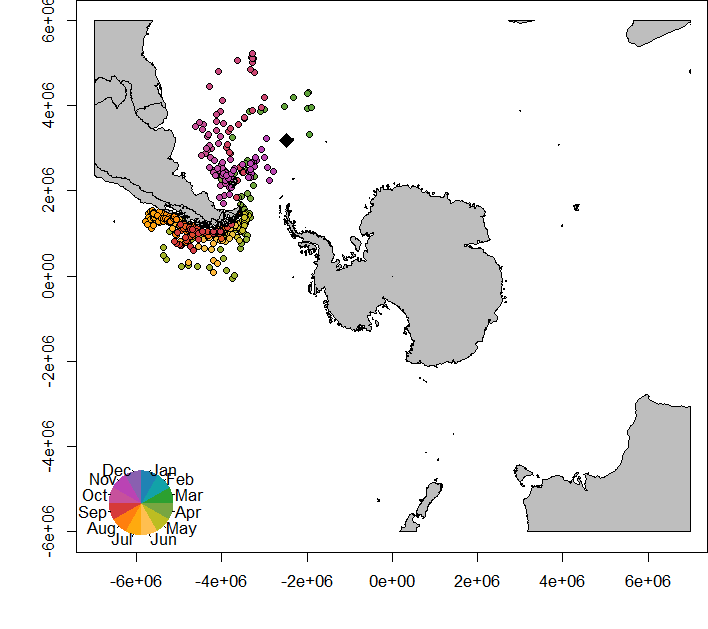


Figure S1. Migration tracks from non-breeding wandering albatrosses tagged with geolocators at Prion Island, South Georgia

Appendix S2: Migration tracks from adult non-breeding wandering albatrosses tagged with geolocators at Bird Island, South Georgia.


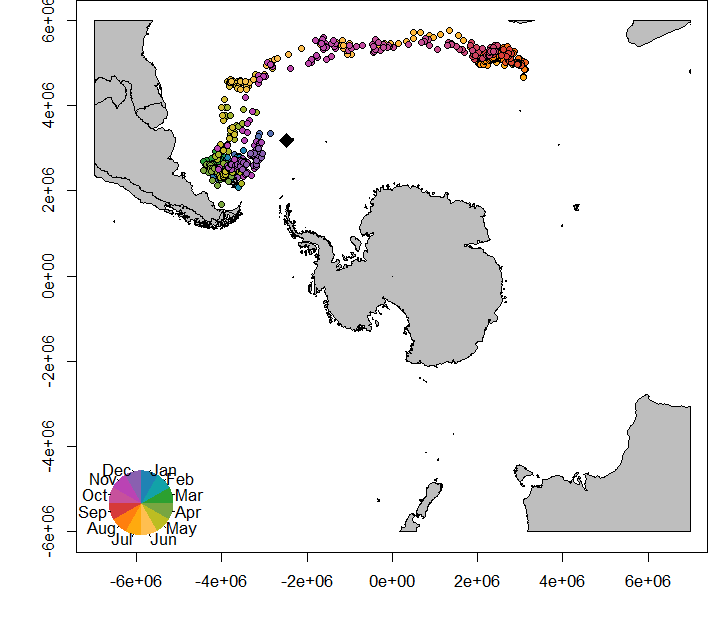

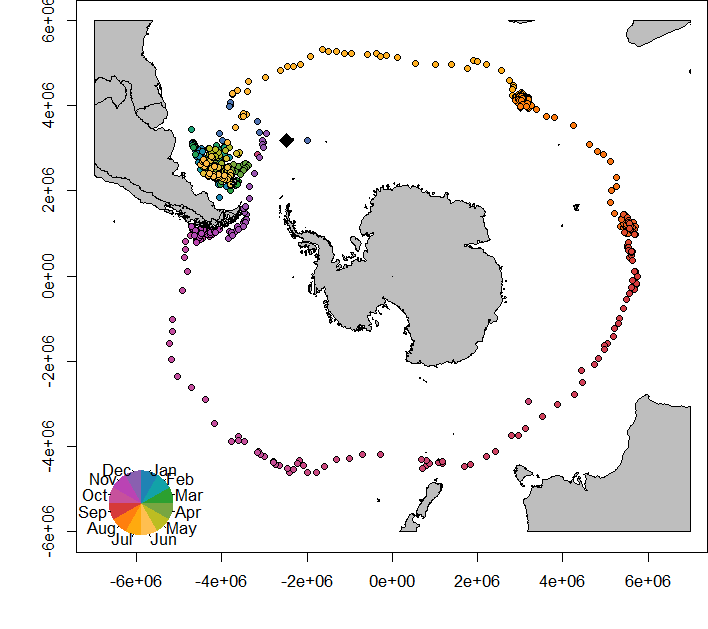

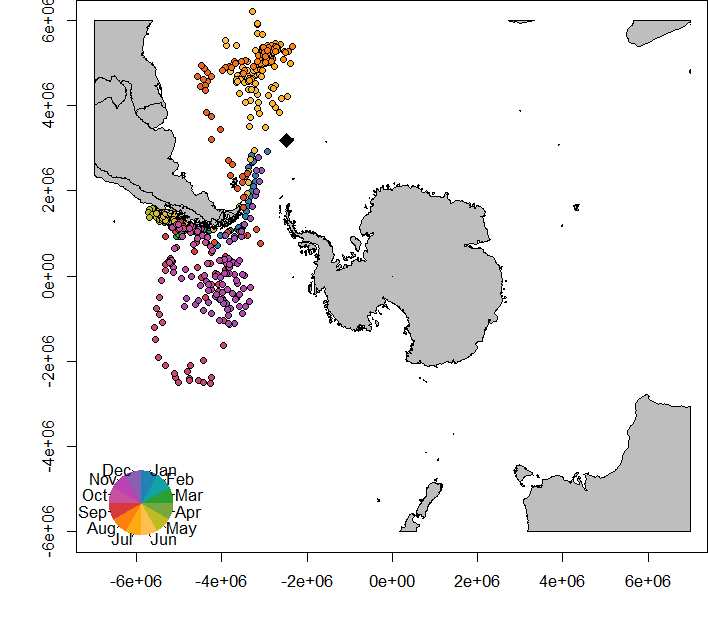

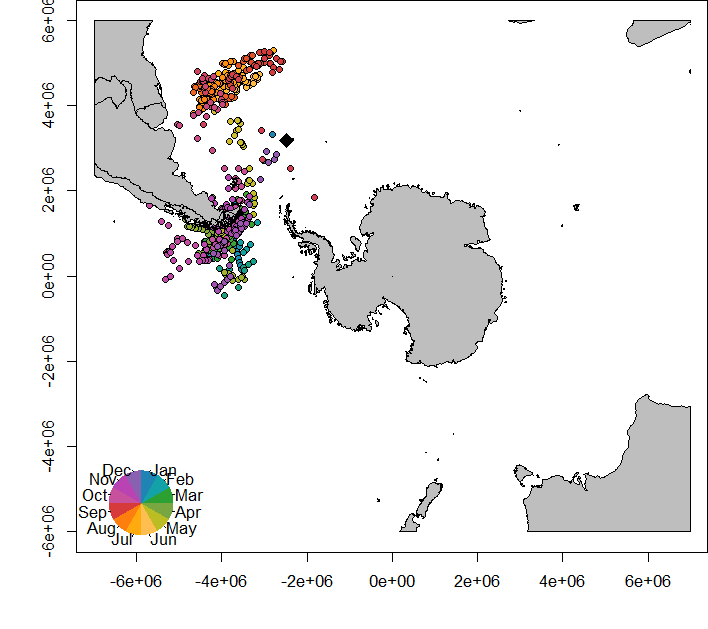

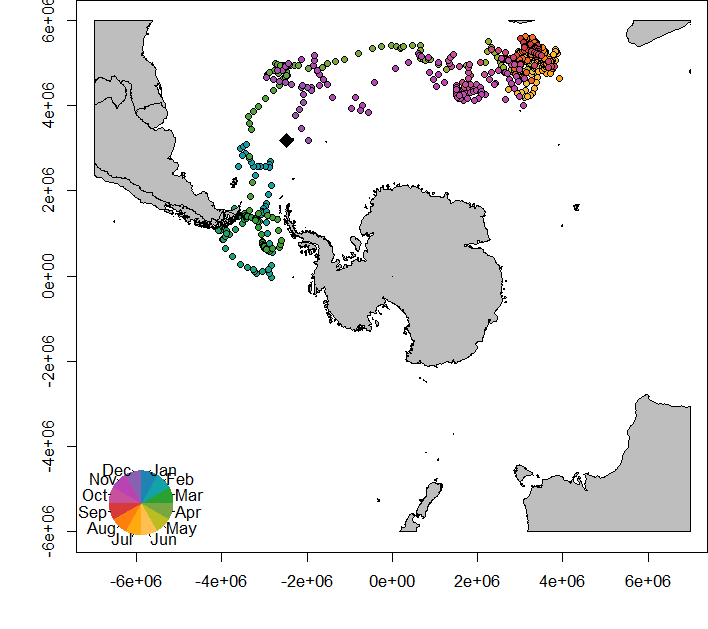

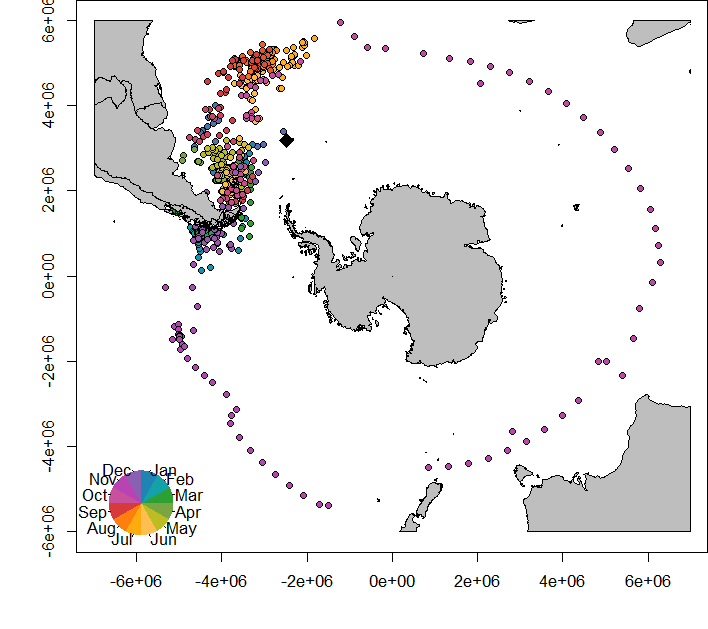

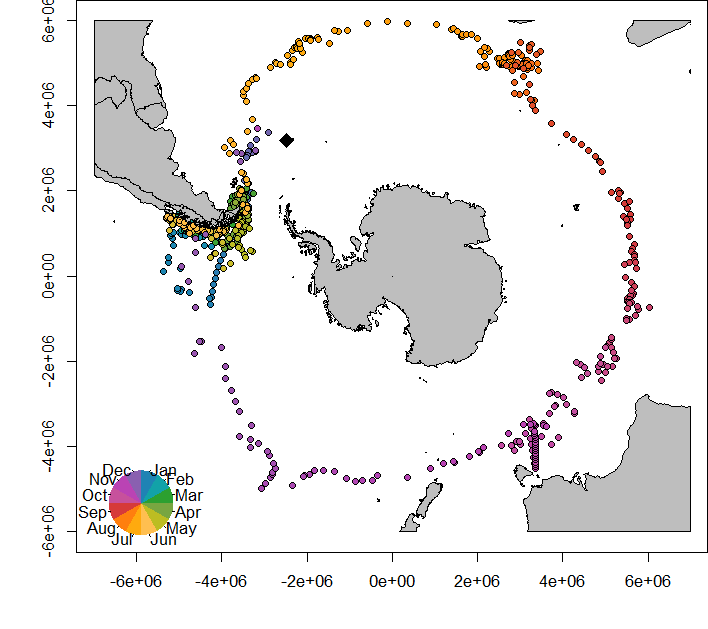

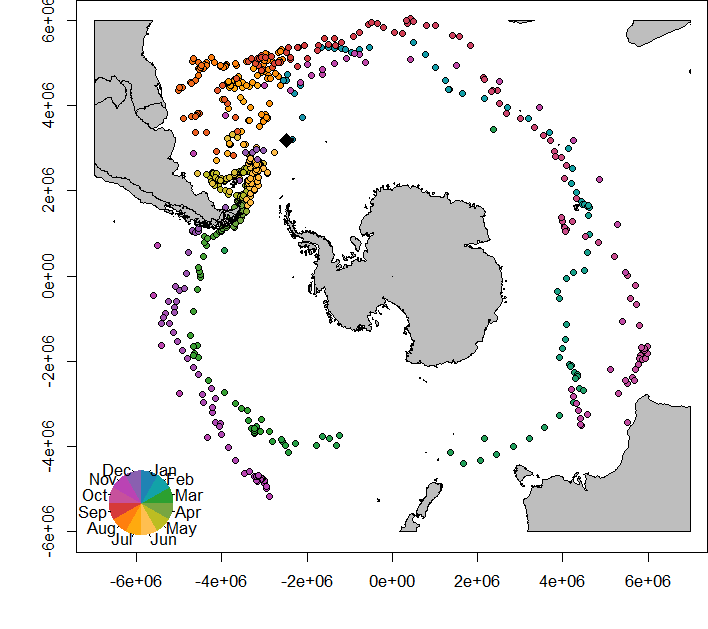

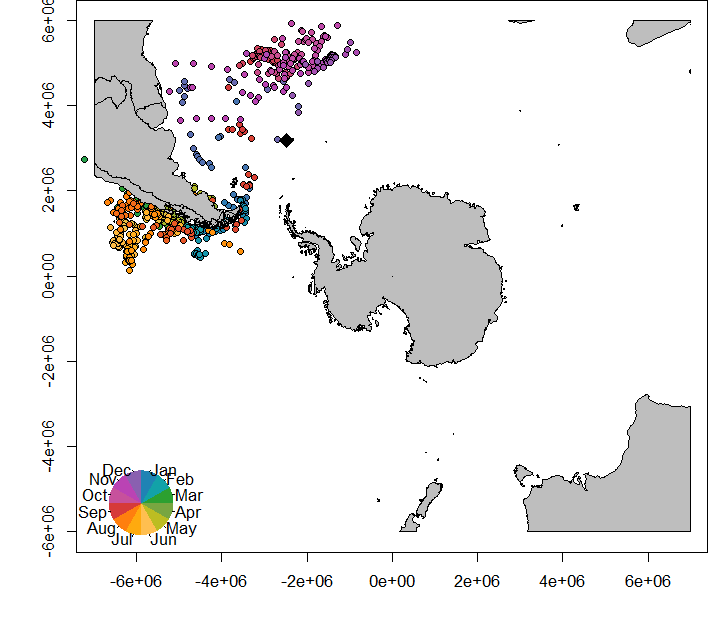

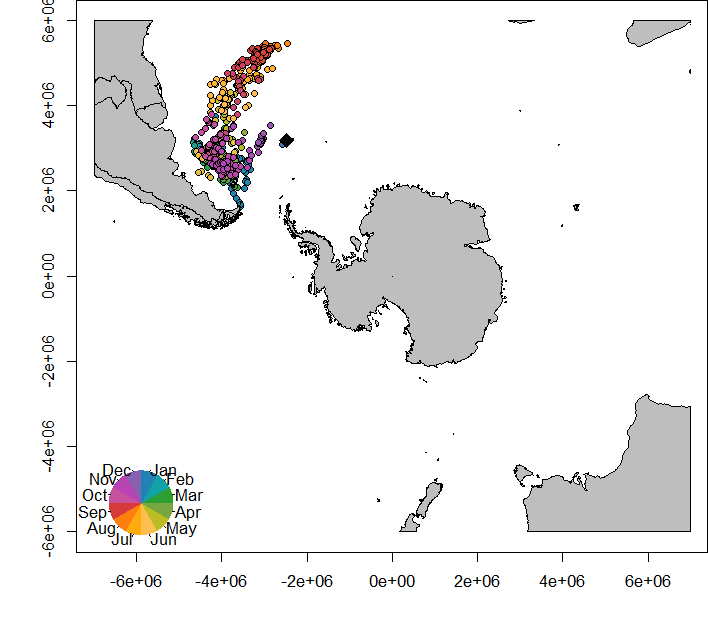

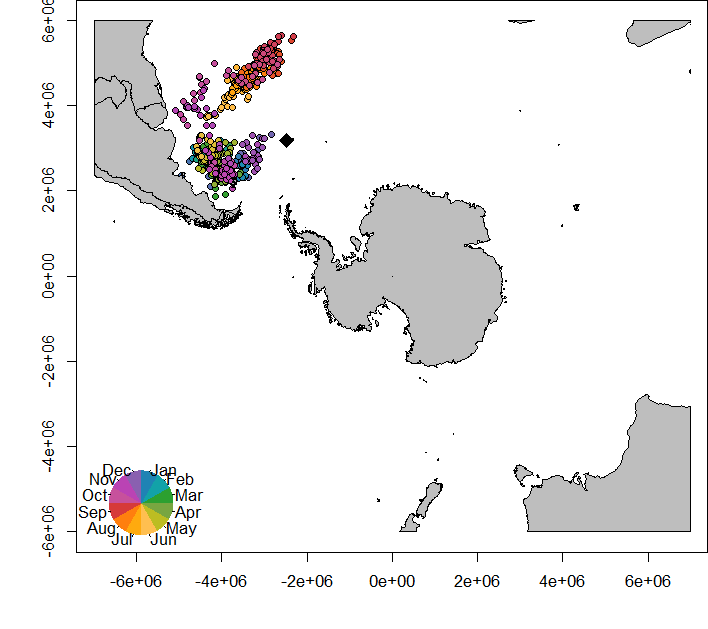

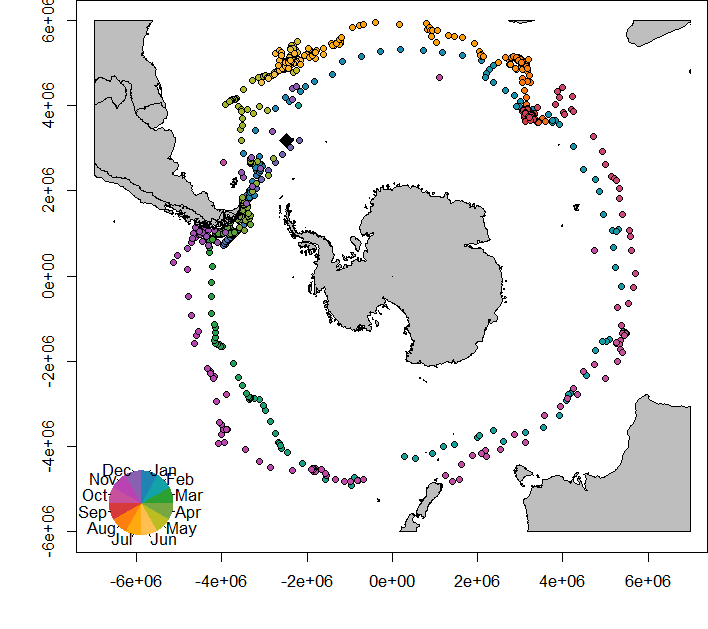


Figure S2. Migration tracks from adult non-breeding wandering albatrosses tagged with geolocators at Bird Island, South Georgia.

Appendix S3. Migration tracks from immature wandering albatrosses tagged with geolocators at Bird Island, South Georgia.


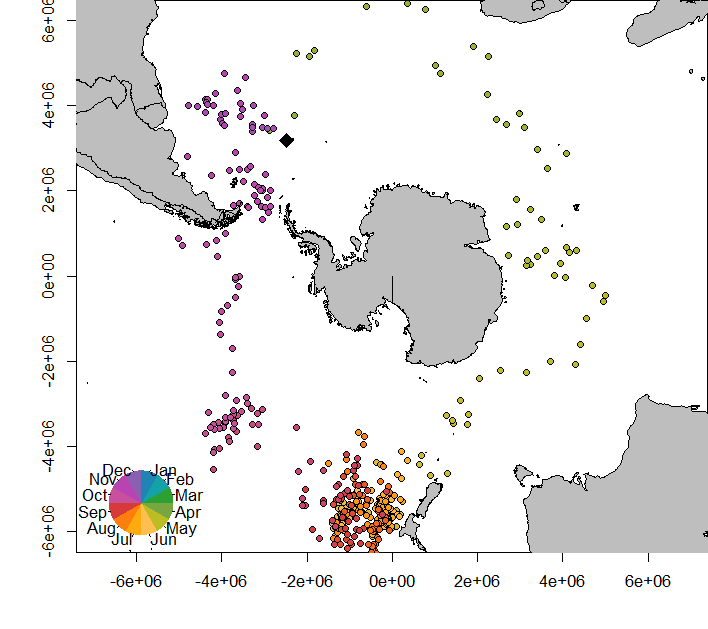

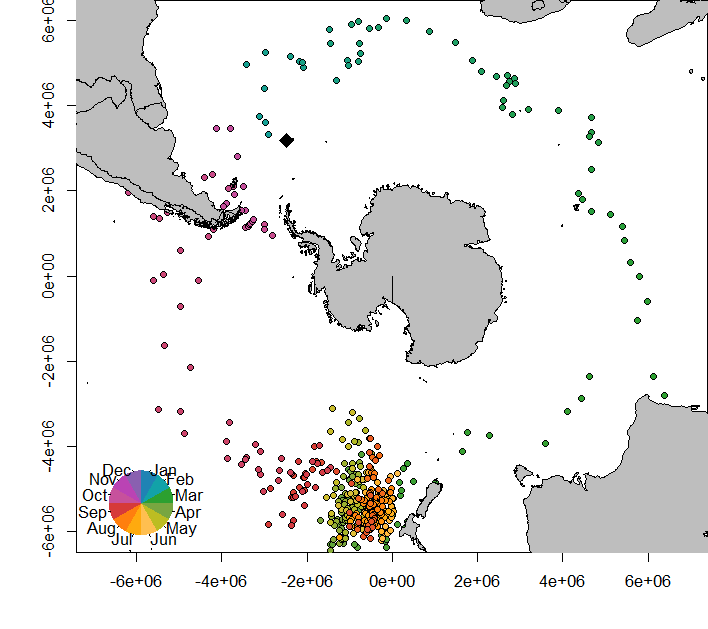

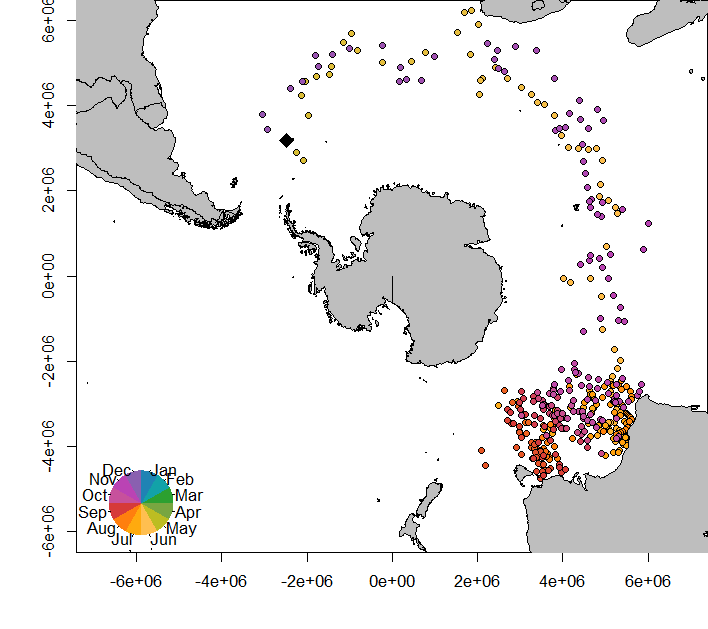

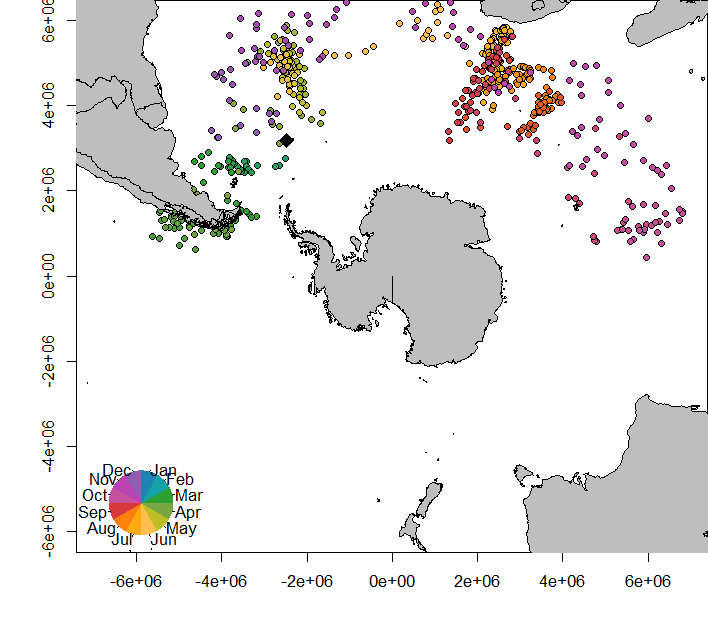

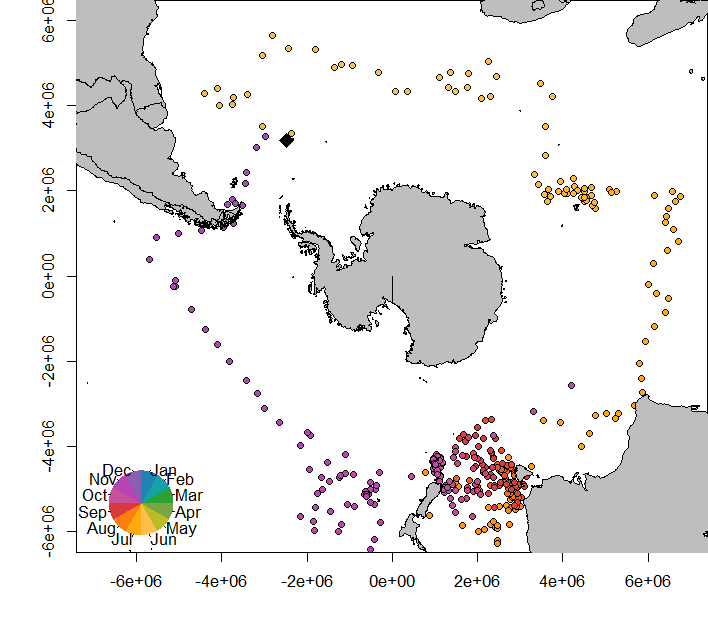

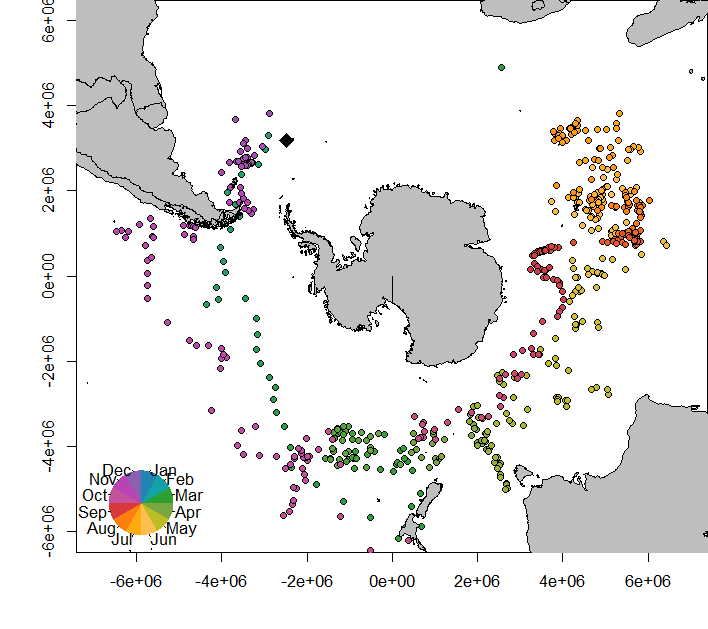

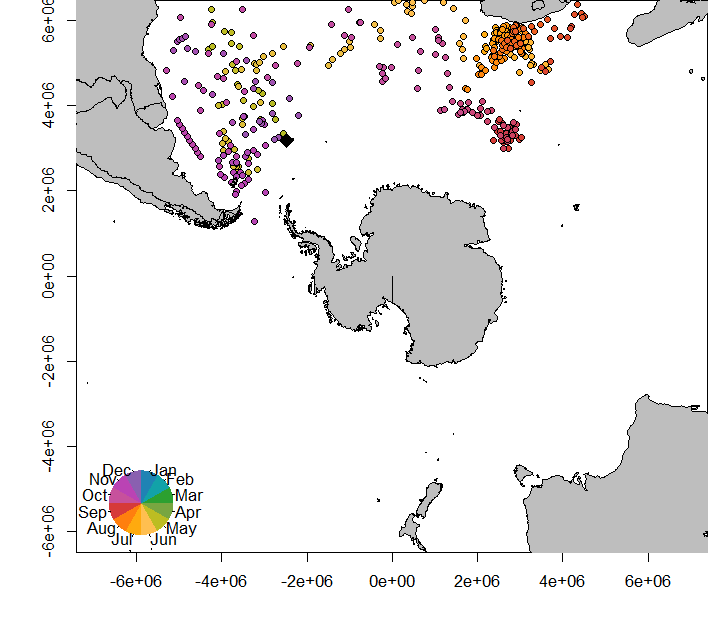

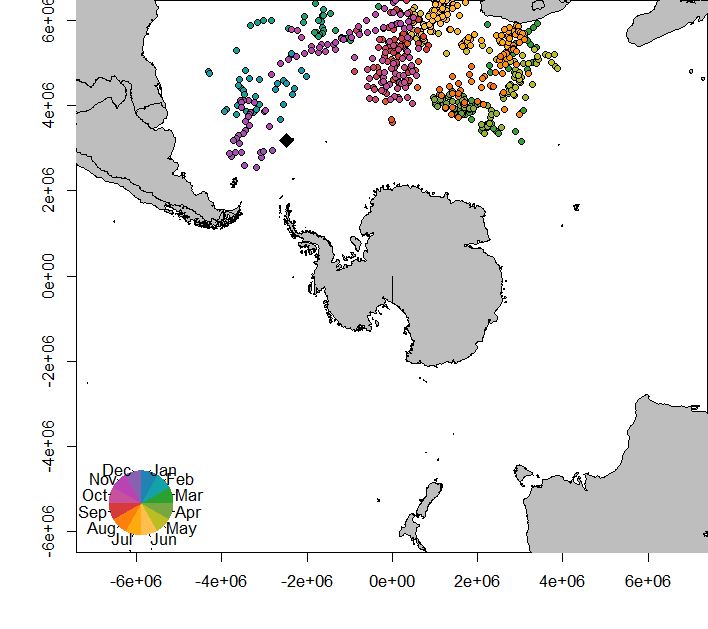

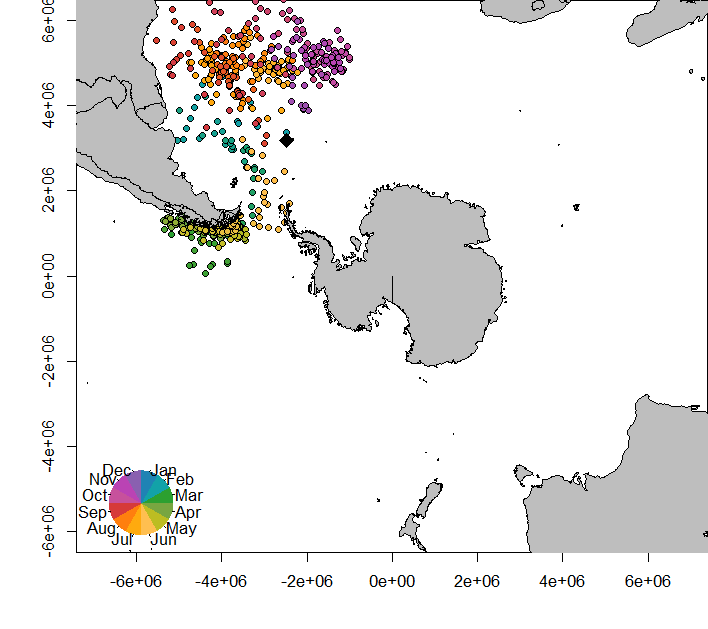

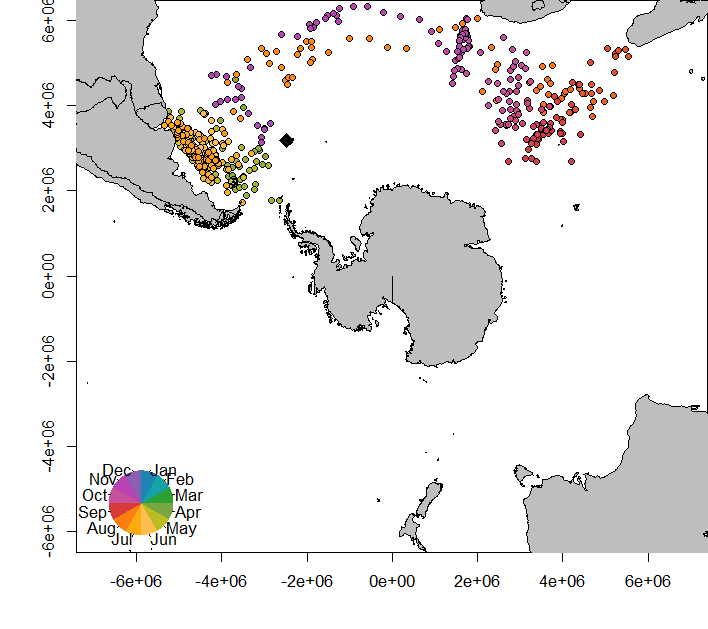

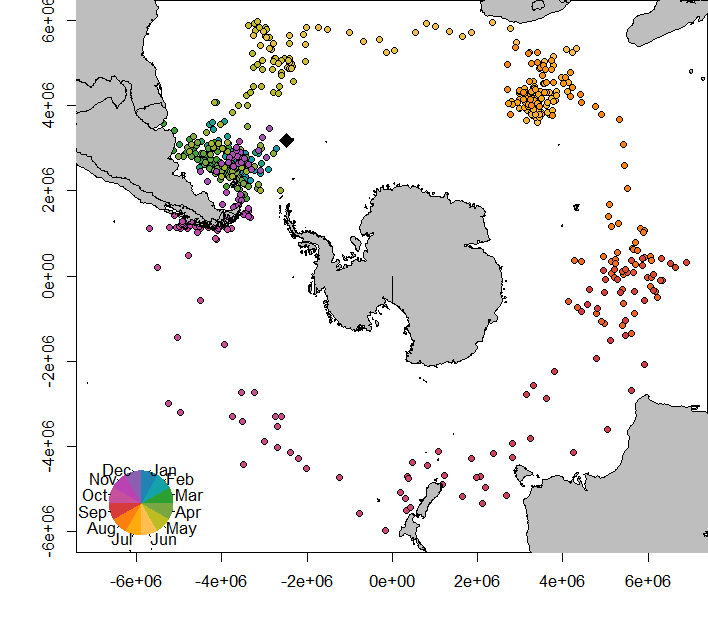

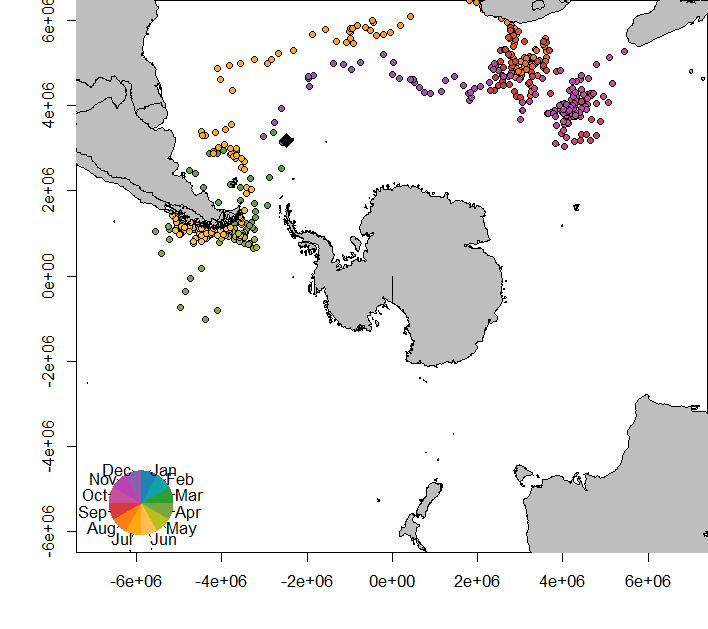

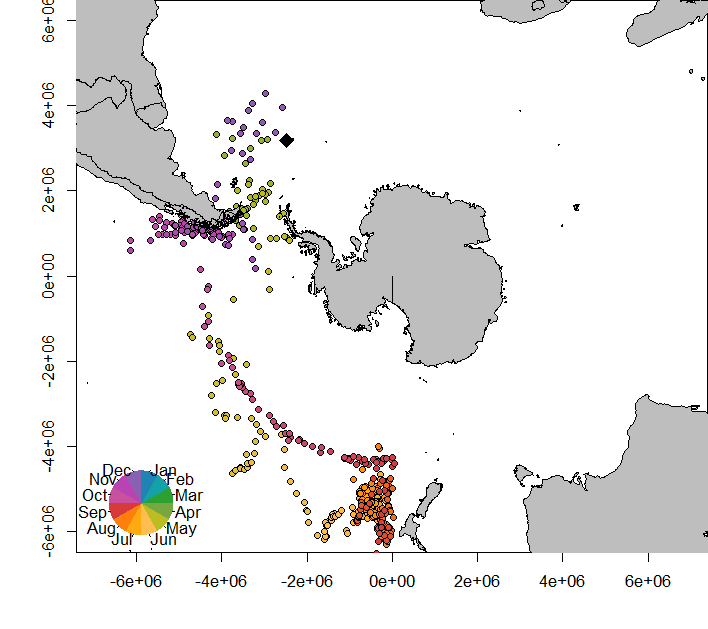

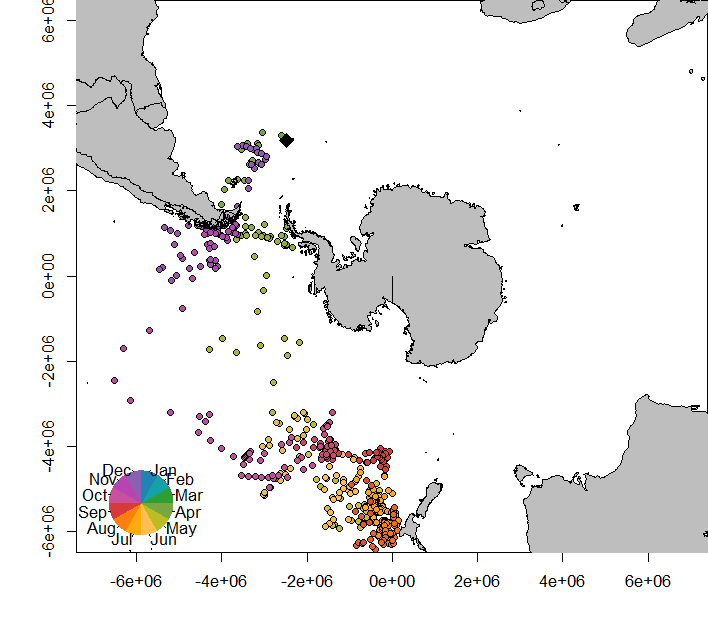

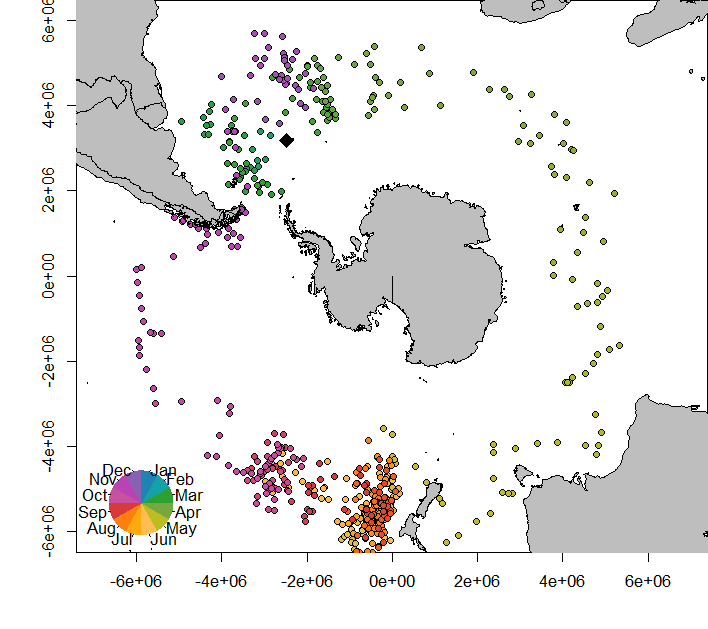

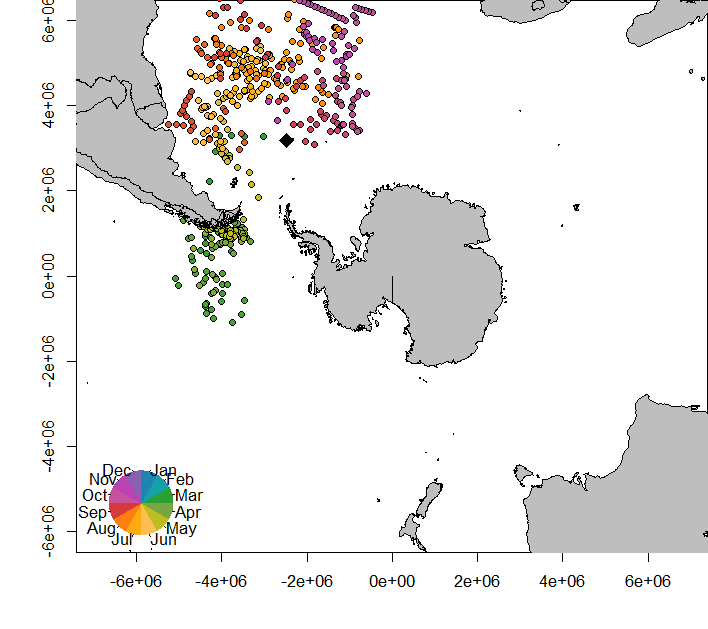

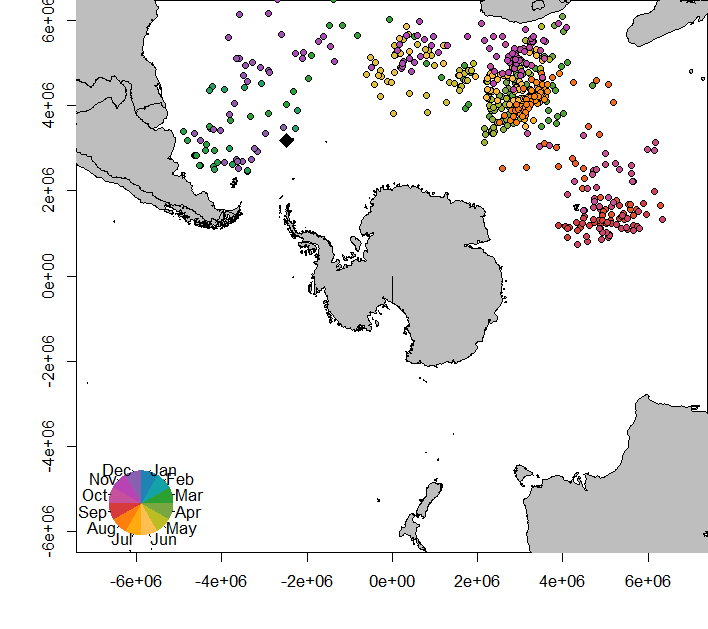

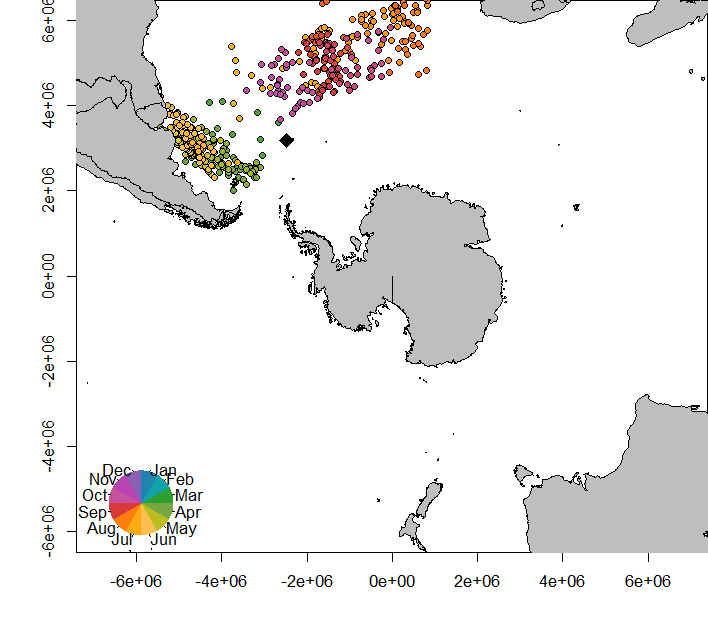


Figure S3. Migration tracks from immature wandering albatrosses tagged with geolocators at Bird Island, South Georgia.

Appendix S4. Individual Utilisation Distributions of wandering albatrosses from South Georgia


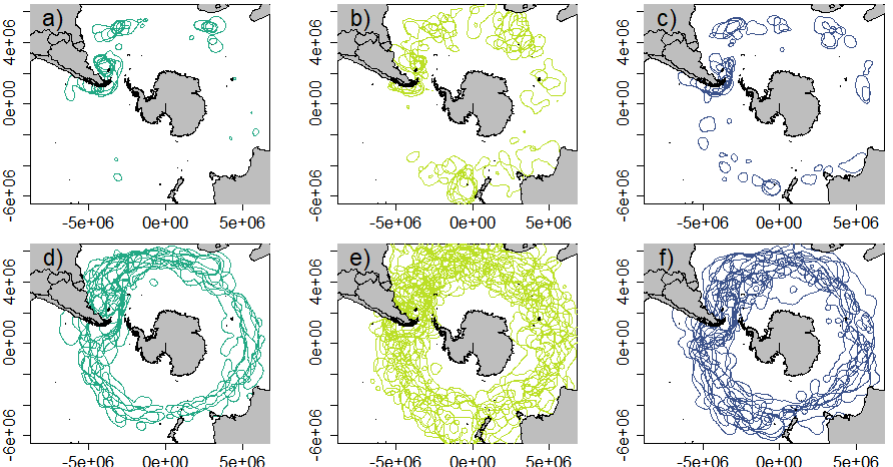


Figure S4. Individual Utilisation Distributions of wandering albatrosses from South Georgia representing the core foraging area (50% UD) of a) Bird Island adults, b) Bird Island immatures, c) Prion Island unknown age, and the home range area (90% UD) of d) Bird Island adults, e) Bird Island immatures, f) Prion Island unknown age. UDs are for individual wandering albatrosses during the non-breeding period.

Appendix S5. Migration strategies adopted by birds tracked from three breeding sites across South Georgia

|  | Circumpolar | Resident | East | West |
| --- | --- | --- | --- | --- |
| Prion Island | 6 | 2 | 2 | 2 |
| Bird Island Adult | 5 | 5 | 2 | 0 |
| Bird Island Immature | 5 | 3 | 7 | 2 |

Appendix S6. Overlap of non-breeding wandering albatrosses from Bird Island and Prion Island, South Georgia, with fishing vessels by flag state and gear type.
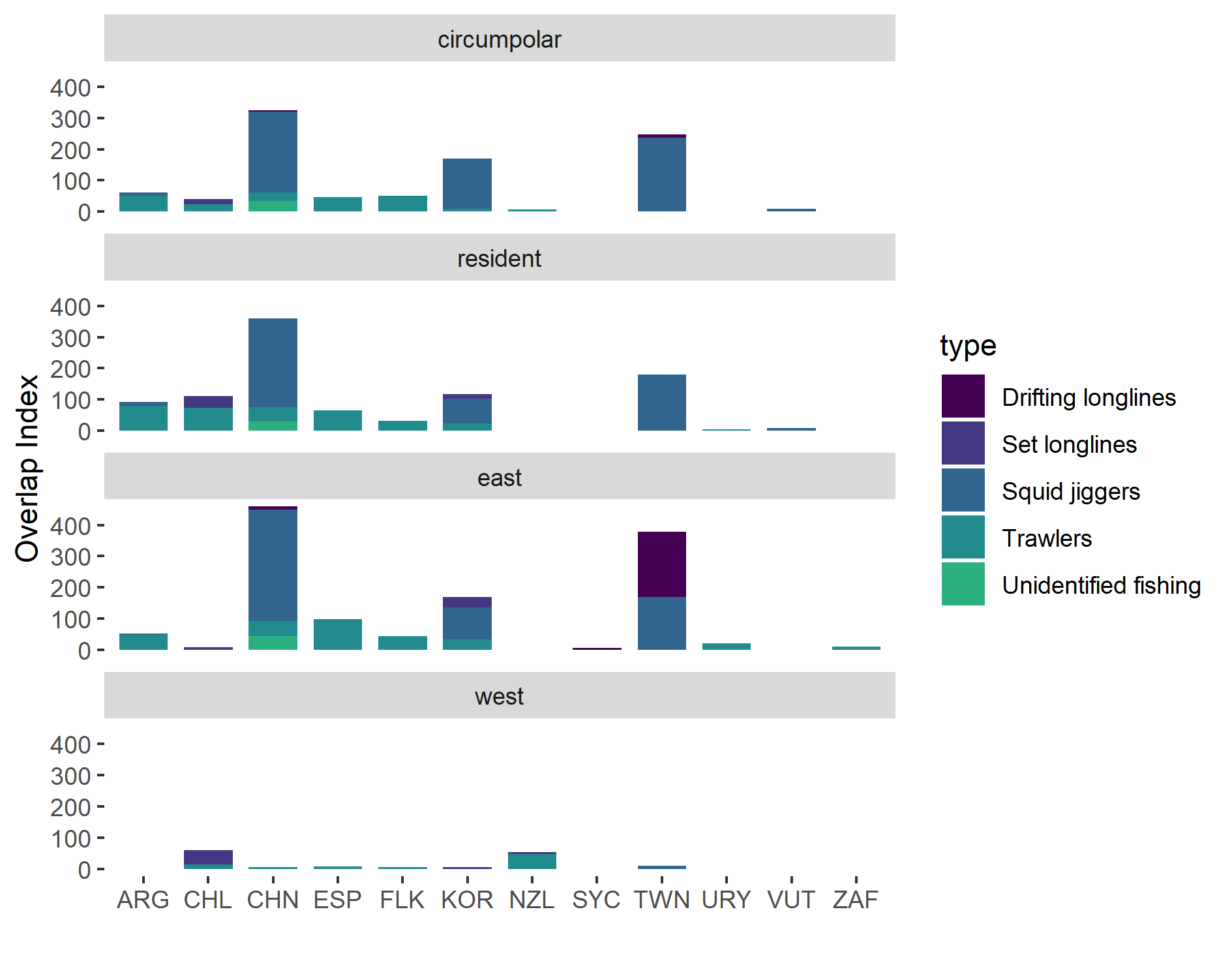


Figure S5. Overlap of non-breeding wandering albatrosses from Bird Island and Prion Island, South Georgia, with fishing vessels by flag state and gear type. Colour scale represents primary fishing gear type for vessels actively fishing, and three letter code the registered flag state. ARG: Argentina, CHL: Chile, CHN: China, ESP: Spain, FLK: Falkland Islands, KOR: South Korea, NZL: New Zealand, SYC: Seychelles, TWN: Taiwan, URY: Uruguay, VUT: Vanuatu, ZAF: South Africa

Appendix S7. Index of the overlap between fisheries and non-breeding wandering albatrosses tracked from Bird Island (BI) in 2006 (immatures) and 2022 (adults) and Prion Island (PI) in 2022, South Georgia, grouped by migration strategy.

|  | All fishing | Drifting (pelagic) longlines | Set (demersal) longlines | Trawlers | Squid jiggers |
| --- | --- | --- | --- | --- | --- |
| Circumpolar | 1142 | 37 | 64 | 279 | 688 |
| Resident | 1166 | 24 | 88 | 382 | 582 |
| East | 1420 | 265 | 70 | 346 | 654 |
| West | 227 | 2 | 77 | 116 | 14 |

Appendix S8. Jurisdictional maps showing the Regional Fisheries Management Organisations


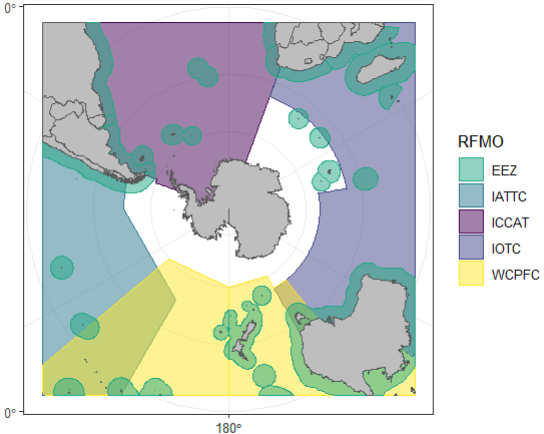

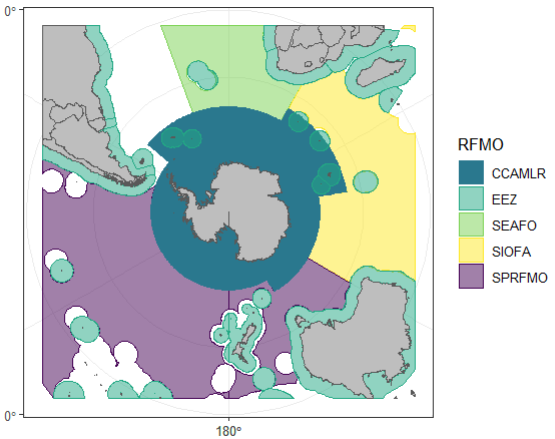


A

B

Figure S6. Jurisdictional maps showing the Regional Fisheries Management Organisation responsible for regulating a) pelagic fisheries (tuna and other billfishes), and b) demersal fisheries across the Southern Ocean. CCAMLR: Commission for the Conservation of Antarctic Marine Living Resources, EEZ: Exclusive Economic Zone, IATTC: Inter-American Tropical Tuna Commission, ICCAT: International Commission for the Conservation of Atlantic Tunas, IOTC: Indian Ocean Tuna Commission, SEAFO: South East Atlantic Fisheries Organisation, SIOFA: Southern Indian Ocean Fisheries Agreement, SPRFMO: South Pacific Regional Fisheries Management Organisation, WCPFC: Western & Central Pacific Fisheries Commission
